# Novel Liposome Eencapsulated Guanosine Di Phosphate based Therapeutic Target against Anemia of Inflammation

**DOI:** 10.1038/s41598-018-35992-2

**Published:** 2018-12-06

**Authors:** Stanzin Angmo, Shilpa Rana, Kamalendra Yadav, Rajat Sandhir, Nitin Kumar Singhal

**Affiliations:** 1 Food Science and Technology Department, National Agri-Food Biotechnology Institute (NABI) Sector-81(Knowledge City), PO Manauli, S.A.S. Nagar, Mohali, 140306 Punjab India; 2 Department of Biochemistry, Panjab University, 160014 Chandigarh, India

## Abstract

Hepcidin, master regulator of iron homeostasis, causes anemia under infectious and inflammatory conditions by reducing intestinal absorption of iron with decreased release of iron from macrophages and liver despite adequate iron stores leading to Anemia of Inflammation (AI). Many therapeutic trials have been carried out but none have been effective due to its adverse effects. In present study, we discover that Guanosine 5’-diphosphate (GDP) encapsulated in lipid vesicle (NH+) was found to inhibit NF-ҝB activation by limiting phosphorylation and degradation of IҝBα, thus, attenuating IL-6 secretion from macrophage cells. Moreover, the suppressed IL-6 levels down regulated JAK2/STAT3 pathway with decrease inflammation-mediated *Hamp* mRNA transcription (HepG2) and increase iron absorption (Caco2) in HepG2/Caco2 co-culture model. Analogous results were obtained in acute and chronic AI mice model thus, correcting haemoglobin level. These results proved NH + GDP as novel therapeutic agent to overcome limitations and suggests it as potential drug to ameliorate AI.

## Introduction

Anemia of Inflammation is the second most prevalent anemia resulting due to the activation of immune response^1,2^. Though being prevalent; it still faces challenges due to poor prognosis and ineffective therapeutic ways. Chronic infection or inflammatory disorders such as rheumatoid arthritis, cancer, chronic kidney diseases and various other inflammatory disorders often results in anemia^3^. Hepcidin, a cysteine rich hepatic peptide hormone plays a crucial role in iron sequestration hindering availability of iron to different organs for cellular functioning. The inflammatory stimulus leads to elevation of hepcidin level which in turn internalize iron exporter ferroportin(FPN) channel resulting in blockage of iron egress from the cells, impairing iron absorption from duodenal intestine and iron recycling inmacrophages^4^. Therefore, it causes iron retention within the cells leading to hypoferremia, resulting in ineffective iron-mediated erythropoiesis. The genetic programming of hepcidin regulation constitutes two major pathway; IL-6/STAT3pathway^5–7^ and bone morphogenetic protein (BMP)/contraction of Sma and Mad (SMAD) pathway^8,9^. Lipopolysaccharide (LPS) produced in gram-negative bacteria cell wall, constitutes major component of bacterial endotoxin. LPS is recognized by Toll-like receptor 4(TLR4) on host cell surface and initiates an inflammatory signalling cascade. Inflammatory pathways up-regulate the expression of TLR4-activated macrophages signal *via* release of various inflammatory factors^10^. This occurs primarily *via* translocation of nuclear factors, such as nuclear factor kappa-light-chain-enhancer of activated B cells (NF-κB) and signal transducer and activator of transcription 3 (STAT3). NF-κB signalling pathways have been considered the classical pathways to modulate the inflammatory response. Once activated, NF-κB translocates into the nucleus to release inflammatory factors. Activation of these pathways regulates oxidative stress response and accelerates inflammatory response^11^. Interestingly, the resident macrophages of liver (Kupffer cells) has found to have no significant role in the up regulation of hepcidin expression in inflammation induced mice models^12–14^.

The previous studies have reported that inflammatory disorders result in the secretion of pro-inflammatory cytokines such as IL-6, which then binds to the IL-6 receptor on the membrane of hepatocytes to activate the JAK2/STAT3 pathway *via* phosphorylation^7^. The phosphorylated STAT3 dimer then translocates to the nucleus, binds to the hepcidin promoter and activates its transcription. Elevated hepcidin eventually binds to the FPN leading to its lysosomal degradation, thus, resulting in intracellular iron accumulation^6^. Hence, transcriptional re-programming of hepcidin could be a novel approach in treating AI symptoms.

It has been reported that Nodakenin, a furanocoumarin glycoside isolated from the dried roots of *A*. *gigas*, has been reported to suppresses LPS-induced inflammatory response in macrophage cell by inhibiting TNF-α and NF-ҝB pathway^15^. Moreover, different strategies were also employed to decrease expression of hepcidin via IL-6/JAK/STAT3 pathway. Hydrogen sulphide^16^, inhibited inflammatory hepcidin by reducing IL-6 levels with silent information regulator 1(SIRT-1)-mediated STAT3 deacetylation. S-propargyl-cysteine^17^, more stable than hydrogen sulfide, suppressed hepatic hepcidin and corrected hypoferremia induced by LPS. Recently, small molecule inhibitors of STAT3 (curcumin, PpYLKTK)^18^, (AG490)^19^ decreases expression of hepcidin by inhibiting the IL-6/STAT3 signalling pathway. AMP-activated protein kinase^20^, as a novel therapeutic target ameliorate AI by promoting suppressor of cytokine signalling 1(SOCS1) mediated JAK2 degradation. However, these approaches are limited with poor pharmacokinetics profile (AG490 and PpYLKT), lack of specificity, stability (STAT3 inhibitors), and competing iron chelating properties (curcumin) with decreased metabolic profile.

GDP is a natural compound and earlier we reported that apart from directly binding and inhibiting hepcidin action, GDP also attenuates inflammation-mediated IL-6/JAK//STAT3-hepcidin axis^21^. However, to explore the mechanistic aspects behind *Hamp* mRNA down regulation *via* IL-6/JAK/STAT3 pathway, we developed a liposomal drug delivery system (NH+) encapsulating GDP (NH + GDP). To enhance the efficacy and stability of GDP on iron availability, GDP was encapsulated within the lipid vesicle having different surface potential. Encapsulated NH + GDP with single positive charge (NH+) was found to be most compatible encapsulating delivery vehicle after all toxological studies. Further, we aimed to investigate the underlying mechanism of NH + GDP on inflammation mediated NF-ҝB activation through IL-6/STAT3 hepcidin axis in *in vitro* and *in vivo* and assessed its therapeutic potential against AI.

## Results

### Physiochemical properties of liposomal formulations

Through lipid extrusion technique, series of liposomal vesicles with different phospholipids composition and net surface charges were prepared. NH+ and NH++ were formed by the cationic phospholipid mixture 1,2-Dioleoyl-sn-glycero-3-phosphocholine (DOPC), 1,2-Dioleoyl-sn-glycero-3 phosphoethanolamine (DOPE) and DOTAP, with single (+) and double (++) positive surface charges respectively. Single and double charge liposomes having different percentage of DOTAP whereas, NH is synthesized without any DOTAP (Table 1). Small lipid vesicles were obtained with a mean size ranging from 116 nm to 154 nm. The zeta potential of liposome’s were obtained in the range of 24–50 mV for cationic lipids (NH+ and NH++), whereas neutral lipids (NH) had a zeta potential of −4 to −6 mV. Encapsulation efficiency of GDP formulation was studied showing cationic charged liposome (NH+) that favour the encapsulation of GDP compound with more than 76.90% efficiency.

**Table 1:** Characterisation of Liposomes GDP.

NAME	LIPOSOME COMPOSTION	CHARGE	POTENTIAL (mV)	SIZE(nm)
NH + GDP	DOPE 60,DOTAP 30,lacPE10 (GDP 10 μM)	+	24–27	138–152
Control NH+	DOPE 60,DOTAP 30,lacPE10	+	25–28	132–154
NH++GDP	DOPE 60,DOTAP 30,lacPE10(GDP 10 μM)	++	45–50	125–126
Control NH++	DOPE 60,DOTAP 30,lacPE10	++	36–44.2	116–117
NH	DOPC45,DOPE45,Cholestrol10(GDP 10 μM)	Neutral	−5 to −6	135–130
Control NH	DOPC45, DOPE45, Cholesterol 10.	Neutral	−4 to −5	135.3–136.7

### Transmission Electron Microscopy (TEM), Scanning Electron Microscopy (SEM) and cellular toxicity of NH+ and NH++ with or without GDP formulation on HepG2 and Caco2 cells

**Figure 1:**
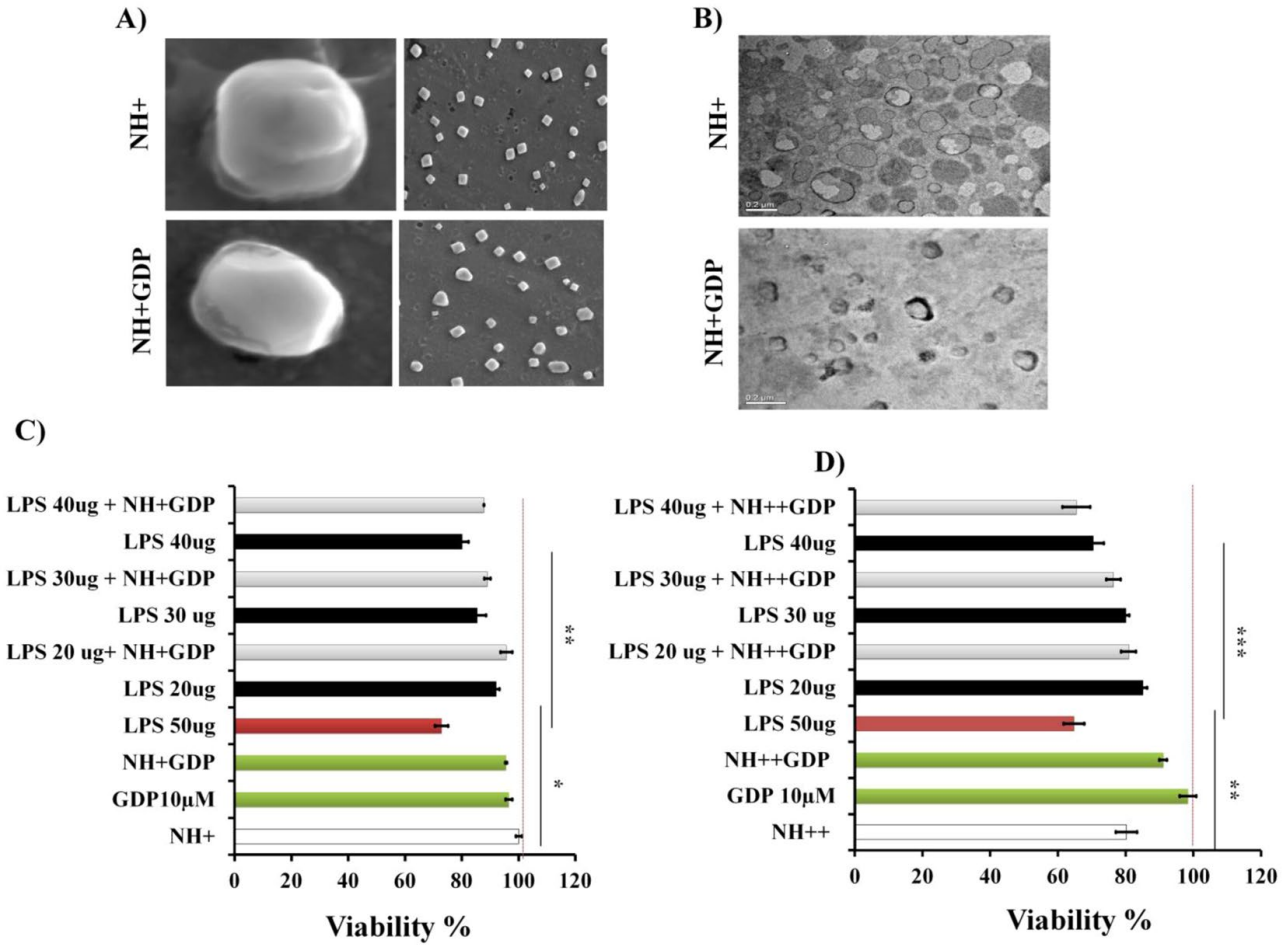
Scanning electron microscopy (SEM), Transmission electron microscopy (TEM) and cytotoxicity of encapsulated NH+ and NH++ GDP formulation on HepG2 cells: A) SEM confirms no significant changes in the structure of the encapsulated NH+GDP as compared to control NH+. B) TEM images indicate unilamilar size of both control NH+ and NH+GDP. C) Viability of HepG2 cells were determined using MTT assay. Cells were treated with encapsulated (NH+GDP and NH++GDP) and control (NH+ and NH++) at indicated LPS concentration. The white bar is control NH+ and NH++ liposome and the green bar is naked GDP and encapsulated NH+GDP, NH++GDP at 10 μM. The indicated red and black bar represent LPS of different concentration (20, 30, 40 and 50 μg) with or without encapsulated NH+GDP and NH++ GDP. Cellular toxicity revealed that NH++ was more toxic at different LPS concentration in a dose dependent manner comparatively to NH+. Non-treated cells were used as 100% viability control (dotted line). Data represent means ± SD of three independent experiments. Differences were analyzed using One-way ANOVA followed by Tukey’s post test. ^*^p < 0.01; ^***^p < 0.001; ^**^p< 0.05

To characterize the physiochemical structure and unilamellar size, SEM and TEM images of NH+ and NH+ GDP were obtained. SEM confirms that there were no significant changes in the structure as compared to control (NH+), indicating that encapsulation of GDP did not cause any distortion in the structure of the NH+ (Fig. 1A). TEM imaging confirms size range of 100 nm for NH+ and NH+ GDP with equal unilamellar size suggesting the integrity of liposome structure after encapsulation (Fig. 1B). *In vitro* MANT-NH + GDP internalization inside HepG2 and Caco-2 cells was validated using blue fluorescence signals (Supplementary Fig. S1). Evaluation of *in vitro* drug release from encapsulated NH + GDP was done by dialysis method correlating with *in vivo* release profile. The *in vitro* release behaviour of the NH + GDP is summarized as the cumulative release percentage as shown in (Supplementary Fig. S2). Cytotoxicity of the liposomal formulations were investigated with both types of liposomes (NH+ and NH++) at different concentration (100, 1000 μg) on HepG2 and Caco2 cell lines using MTT assay. NH++ relatively showed higher cytotoxicity than NH+ at indicated concentrations may be due to cellular membrane damage by NH++ vesicles to a greater extent. Furthermore, the percentages of viable cells were more when treated with NH+ as compared to NH++ liposomes (Supplementary Fig. S3). Additionally, a dose response experiment was conducted with NH+ and NH++ formulation with and without GDP at indicated concentration of LPS on HepG2 cells. The cell viability assay showed that NH + GDP at dose dependent concentration of LPS showed more cell viability compared to NH++ GDP, confirming higher efficacy and reduced cytotoxicity. In agreement, we choose the less toxic NH+ formulation (DOPE60, DOTAP30, lacPE10) as ideal liposome composition to encapsulate the GDP (10 μM) for further studies (Fig. 1C,D).

### NH + GDP suppresses LPS-induced pro-inflammatory cytokines production and NF-ҝB activation in PMA-differentiated U937 monocytic cell line

**Figure 2:**
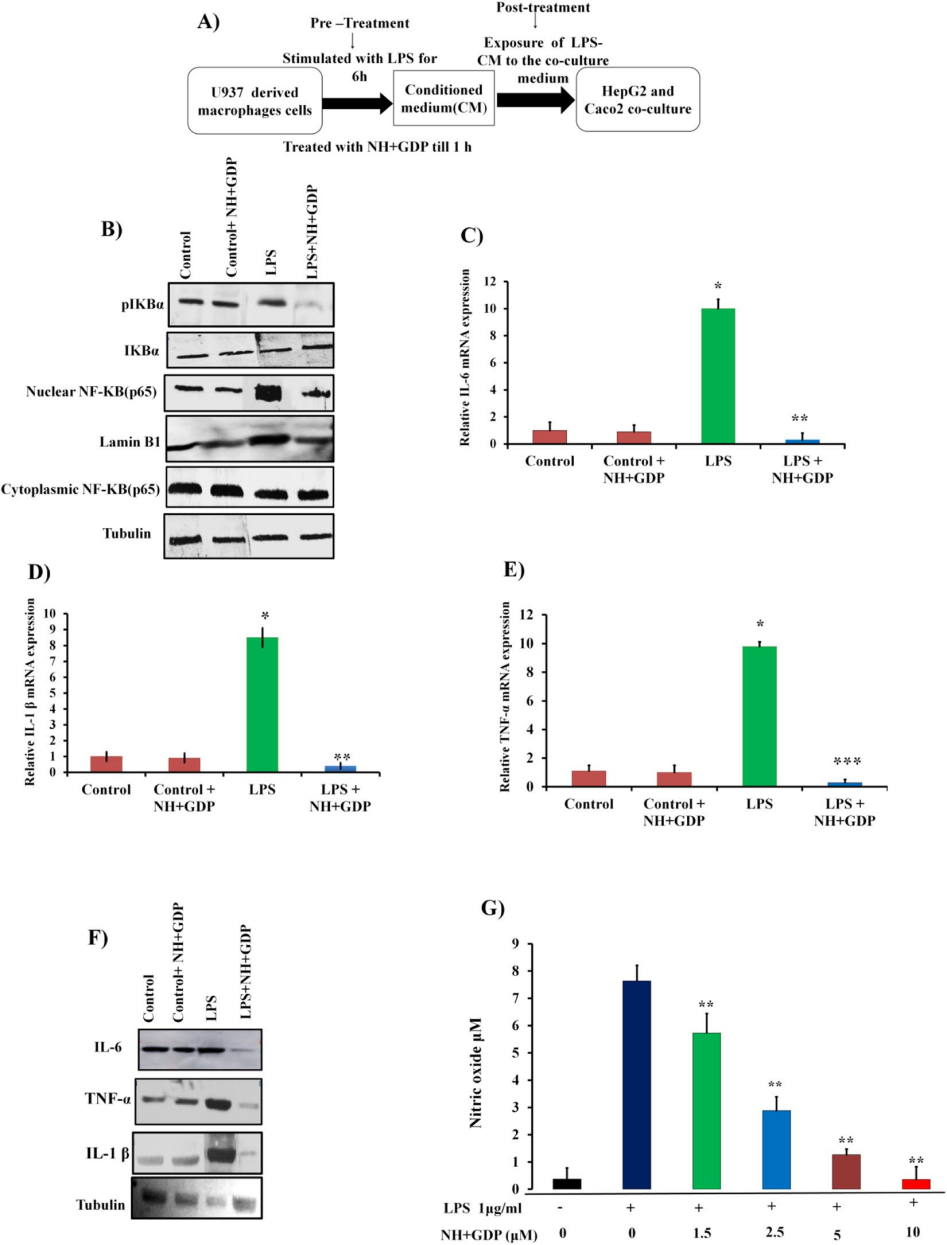
NH+GDP suppresses NF*-ҝB* pathway reducing pro-inflammatory cytokines release (**A**) Flowchart representation of CM model of U937 cells were treated with NH+GDP 1h before LPS and further the LPS-CM was exposed to HepG2 and Caco2 co-culture cells. (**B**) NH+GDP decreases phosphorylation of IκB-α that prevents the nuclear translocation of the p65 subunit of NF-κB from cytosol into the nucleus as compared to the LPS group. (**C–E**) NH+GDP significantly decreases the mRNA expression of IL-6, TNF-α and IL-1β pro-inflammatory cytokinesin U937 macrophage whole cell lysate. (**F**) Treatment with NH+GDP significantly decreases protein expression of IL-6, TNF-α and IL-1β in the whole cell lysate. (**G**) Reduced NO level in whole cell lysate was observed with NH+GDP treatment in dose dependent manner. Full blots are presented in supplementary Figure S11. *p* values were calculated using one-way ANOVA. **p ≤ 0.05. Densitometry analysis of represented immunoblot was demonstrated in supplementary Figure S6. Tubulin was used as an internal control. Data are presented as the mean+SD of three individuals experiments. *p* values were calculated using one-way ANOVA. **p ≤ 0.05,***p ≤ 0.001. Differences were analyzed using One-way ANOVA followed by Tukey’s post test. *^,#^*p <* 0.01; ***^, **##**^*p <* 0.001, *p <* 0.05.

We used CM of PMA-induced differentiated U937 monocytic cells and co-culture model consisting of HepG2 and Caco2 cells to mimic the pathophysiological conditions *in vivo*. Earlier studies reported that LPS induced inflammation increases *Hamp* mRNA expression in HepG2 and macrophages cells^22^. Time course assessments suggest 6 h after LPS treatment as the peak point of hepcidin mRNA induction in HepG2 cells (Supplementary Fig. S4). The macrophage cells were stimulated with LPS for 6 h and pre-treated with NH + GDP for 1 h (termed as pre-treatment) followed by exposure of LPS-CM to the HepG2/Caco2 co-culture model, which was termed as “post treatment” (Fig. 2A).

Initially, we investigated the effect of GDP and NH + GDP on IL-6 level in U937 derived macrophage cells. LPS-induced inflammation increases IL-6 secretion through TLR-4 mediated NF-ҝB activation in macrophages cells^10,23^. Additionally, in comparison to free GDP, encapsulated GDP(NH + GDP) was more effective in decreasing IL-6 in dose dependent concentration. This assay marked compelling evidence that encapsulated NH + GDP was more effective in reducing inflammation-mediated IL-6 secretion as compared to non-encapsulated GDP (Supplementary Fig. S5). To explore the mechanistic action of NH + GDP, U937 derived macrophage cell lines were stimulated with LPS to activate the NF-κB pathway. Inactive state of NF-κB binds to its inhibitor protein, IκB-α, in the cytoplasm, but on cellular stimulation IκB-α is phosphorylated at specific serine residues and undergoes polyubiquitination and protoesomal degradation, which releases NF-κB subunit, allowing it to be translocated to the nucleus^23^. Interestingly, we found that NH + GDP attenuates phosphorylation of IҝB-α and NF-ҝB as shown in (Fig. 2B) that prevents the nuclear translocation of the p65 subunit of NF-κB, thus decreasing the induction of pro-inflammatory cytokines. This decrease was paralleled with reduced transcription of pro-inflammatory cytokines (IL-6, TNF-α and IL-1β) as well as decreased protein expression of cytokines (Fig. 2C–F) along with suppressed NO production (Fig. 2G) in macrophage whole cell lysate in dose dependent manner. These *in vitro* results indicate that NH + GDP inhibit LPS-induced phosphorylation and degradation of IκB-α thus, decreasing levels of pro-inflammatory cytokines.

### NH + GDP down regulates IL-6/JAK/STAT3 pathway suppressing hepcidin expression

**Figure 3:**
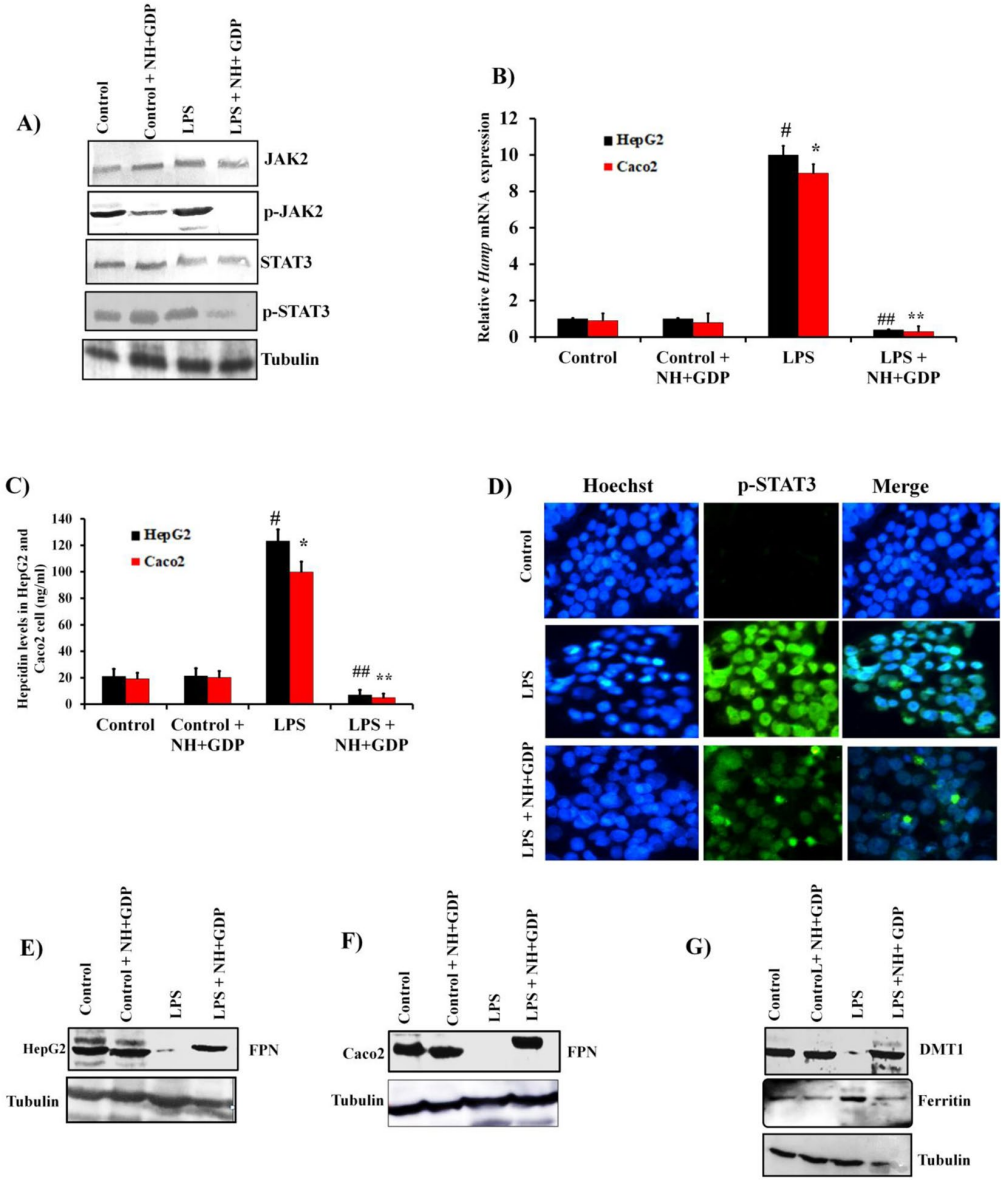
Target specific NH+GDP inhibit *Hamp* mRNA expression by decreasing IL-6 secretion in HepG2 and Caco2 co-culture model: (**A**) NH+GDP inhibited LPS-CM-induced JAK2/STAT3 activation. (**B,C**) Decrease in *Hamp* mRNA and hepcidin concentration was observed as compared to LPS treated group in HepG2 and Caco2 co-culture cells. (**D**) Immunofluorescence images clearly indicate that NH+GDP suppressed pSTAT3 nuclear translocation induced by IL-6. (**E,F**) Increase FPN protein expression revealed that NH+GDP prevents hepcidin-induced internalization of FPN in HepG2 and Caco2 co-culture cells. (**F**) LPS-mediated inflammation decreases iron uptake from DMT1 transporter whereas, NH+GDP treatment reverses this effect with increase cellular iron uptake and reduced iron storage ferritin in Caco2 cells. Densitometry analysis of represented immunoblot was demonstrated in Supplementary Figure S7. Full blots are presented in Supplementary Figure S12. Tubulin was used as a internal control. Data were normalized to mRNA expression of a housekeeping gene, *GAPDH*. Data are presented as the mean+SD of three individual’s experiments. *p* values were calculated using one-way ANOVA. ‘*’ with *p* ≤ 0.01 control *vs* LPS and ‘**’ with *p* ≤ 0.05 LPS+NH+GDP *vs* LPS vs. ‘^#^’ with *p* ≤ 0.01 control *vs* LPS and ‘^##^’ with *p* ≤ 0.05 LPS+NH+GDP *vs* LPS.

In agreement with the decreased IL-6 levels in LPS-CM derived after NH + GDP treatment, subsequently there was reduced binding of IL-6 to IL-6 receptor attenuating the activation of JAK2/STAT3 pathway responsible for *Hamp* mRNA transcription in HepG2 cells. Consistent results were observed with decreased phosphorylation of pJAK2 and pSTAT3 (Fig. 3A) thus, reducing *Hamp* mRNA and hepcidin level (Fig. 3B,C). The measurement of hepcidin indicates significant difference in LPS+ NH+ GDP treated group in both HepG2 and Caco2 cells. The mean hepcidin concentration in LPS+ NH+ GDP (12 ± 1.2 ng/ml HepG2), (8.2 ± 1.1 ng/ml Caco2) showed significant decrease in hepcidin level as compared to LPS treated group(120 ± 2.0 ng/ml HepG2), (101 ± 1.5 ng/ml Caco2). Consistent results were observed with decreased phosphorylation of pJAK2 and pSTAT3 (Fig. 3A) thus, reducing *Hamp* mRNA and hepcidin level (Fig. 3B,C). The measurement of hepcidin indicates significant difference in LPS + NH + GDP treated group in both HepG2 and Caco2 cells. The mean hepcidin concentration in LPS + NH + GDP (12 ± 1.2 ng/ml HepG2), (8.2 ± 1.1 ng/ml Caco2) showed significant decrease in hepcidin level as compared to LPS treated group(120 ± 2.0 ng/ml HepG2), 99 ± 1.0 ng/ml Caco2). Immunoblot analysis indicated that LPS increased the translocation of pSTAT3 into the nucleus due to the activation of JAK/STAT3 pathway, whereas NH + GDP treatment down regulated JAK/STAT3 pathway and hence decreased translocation of pSTAT3 into the nucleus (Fig. 3D). Concomitantly, FPN protein expression decreased due to hepcidin-mediated internalization of FPN. However, NH + GDP reversed this effect in HepG2 and Caco2 cells (Fig. 3E,F). As expected, LPS-induced inflammation decreased DMT1 iron transporter expression in Caco2 cells (Fig. 3G) due to hepcidin-mediated proteasome internalisation of DMT1. Whereas, NH + GDP treatment up regulated the DMT1 expression across the apical membrane in comparison to LPS treated group. Similarly, increase iron storage ferritin level was observed in LPS-induced co-culture model due to inflammation mediated FPN internalization, thus, restricting iron release. However, NH + GDP treatment reverses this effect with decrease iron accumulation thus restricting iron storage ferritin level in Caco2 cells^24^ (Fig. 3G). To investigate whether IL-6 stimulant effects the hepcidin expression in HepG2 monoculture cells and FPN expression in Caco2 cells, we used IL-6 as a stimulant in HepG2 and Caco2 mono-culture cells for evaluating the hepcidin and FPN expression respectively. The results showed that in HepG2 mono-culture cells, the IL-6 stimulant increased the expression of hepcidin as IL-6 acts an inducer of JAK/STAT3 pathway which further increases the hepcidin expression. On the other hand, IL-6 has no effect on FPN expression in Caco2 mono-culture cells as FPN degradation is dependent on hepcidin and not on IL-6 alone (Supplementary Fig. S10).

### NH + GDP suppresses expression of pro-inflammatory mediators and IL-6/JAK/STAT3 pathway in acute mice model

**Figure 4:**
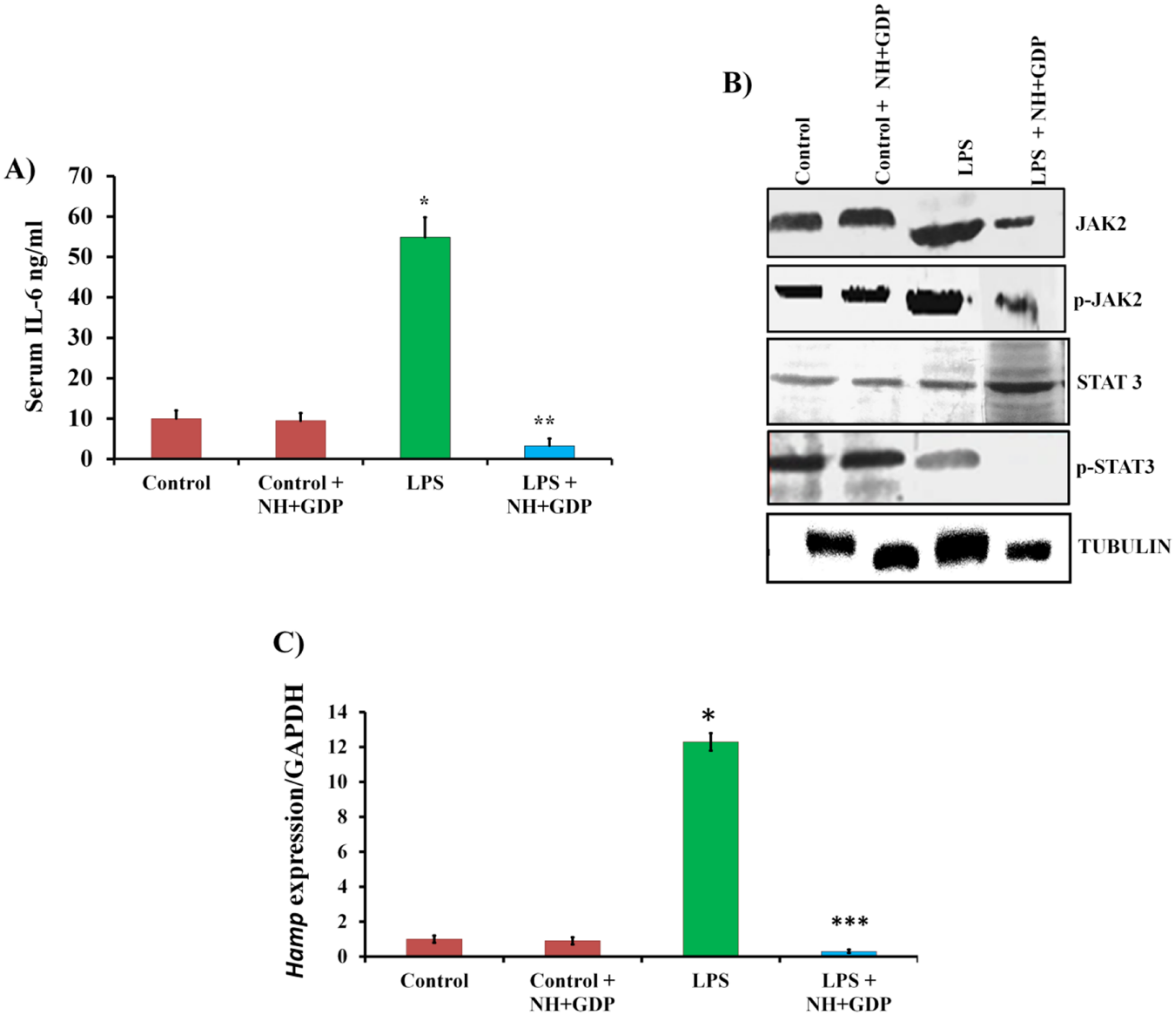
NH+GDP suppresses LPS–induced *Hamp* expression in acute model: (**A**,**B**) NH+GDP significantly reduced serum IL-6 level suppressing the phosphorylation of JAK2/STAT3 pathway in hepatocytes. Densitometry analysis of represented immunoblot was demonstrated in supplementary Figure S8.Tubulin was used as a internal control. Full blots are presented in Supplementary Figure S13.(**C**) Consistently NH+GDP decreases LPS-mediated *Hamp* mRNA expression relieving LPS-induced inflammation in acute mice models. Results are normalized to *GAPDH* and expressed as mean ± SD for *n* animals (n = 8/group). *p* values were calculated using one-way ANOVA. ***p ≤ 0.001 LPS+NH+GDP *vs* LPS **p ≤ 0.05 LPS+NH+GDP *vs* LPS and *p ≤ 0.01 Control *vs* LPS.

*In vitro* studies provided clear evidence that NH + GDP attenuates NF-ҝB pathway activation thus, reducing pro-inflammatory mediators (IL-6, TNF-α and IL-1β) level. In correspondence, subsequently there was reduced binding of IL-6 to its receptor to down regulate IL-6/JAK/STAT3 pathway with decreased *Hamp* mRNA transcription. LPS-induced inflammation increases pro-inflammatory cytokines production (IL-6) with increase transcription of *Hamp* mRNA level leading to hypoferremia^25^. To elucidate the IL-6/STAT3 pathway involved in hepcidin expression BALB/c mice were challenged with LPS (IP) for 6 h followed by NH + GDP (IP) treatment for 30 minutes. In association, we found significant decrease in serum IL-6 (Fig. 4A) with decreased phosphorylation of JAK2 and STAT3 activation thus, reducing transcription of *Hamp* mRNA level in liver tissue (Fig. 4B,C). These results indicate that NH + GDP attenuate hepcidin expression in liver through suppressing pro-inflammatory IL-6 levels by down regulating IL-6 and JAK/STAT3 activation.

### NH + GDP reduces *Hamp* mRNA expression by suppressing IL-6/STAT3 pathway in chronic AI model

**Figure 5:**
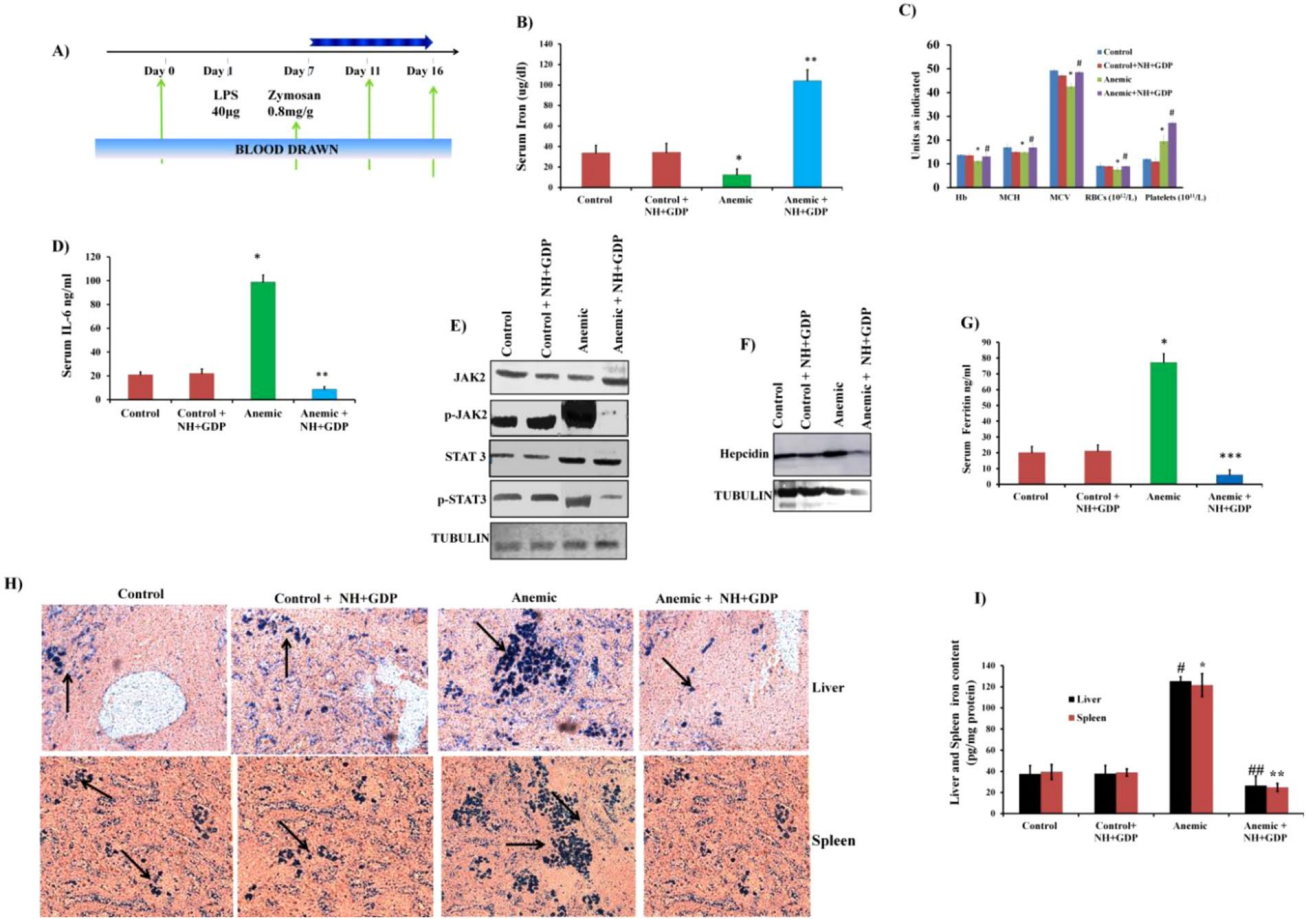
NH+GDP ameliorates AI in chronic model thus, maintaining normal iron homeostasis: (**A**) Diagrammatic representation of dose interval and time progression towards anemia. (**B**) Elevated serum iron concentration was observed with NH+GDP treated group. (**C**) Complete blood count (CBC) indices of mice injected (I.P) with normal saline or LPS+Zymosan and treated with NH+GDP (*i*.*p*. i.e. 30 mg/kg body) after 28 days. (**D**) NH+GDP significantly attenuates serum IL-6 level evoked by LPS+Zymosan induced inflammation. (**E**,**F**) NH+GDP suppressed phosphorylation of JAK2/STAT3 thus, decreasing inflammatory hepcidin-25 protein expression; tubulin was used as an internal control. Densitometry analysis of represented immunoblot was demonstrated in Supplementary Figure S9. Full blots are presented in Supplementary Figure S13.Tubulin was used as a internal control. (**G**) In parallel, significant decrease in serum ferritin level was observed in NH+GDP treated group indicating effective iron egress for erythropoiesis. (**H**) Liver and splenic iron level indicates decrease in iron content level after treatment with NH+GDP. (**I**) Increased iron deposit was observed in anemic state, whereas NH+GDP reversed this effect with decrease iron accumulation in liver and spleen. Results are normalized to *GAPDH* and expressed relative to controls. *n* = 8/group. *p* values were calculated using One-way ANOVA. ‘*’ with *p* ≤ 0.01 control *vs* anemic, ‘**’*p*≤ 0.05 NH+GDP *vs* anemic, ‘***’*p*≤ 0.001 NH+GDP *vs* anemic.

To induce the chronic AI model, BALB/c mice were challenged with LPS (IP) on the first day followed by Zymosan (IP) a week later and then sacrificed after 10 days as depicted in (Fig. 5A). Next AI mice were treated with NH + GDP (IP) every 24 h for 2 weeks. Treatment with NH + GDP significantly increases serum iron concentration (Fig. 5B) with rise in haemoglobin level and erythrocyte number thus, correcting inflammation-induced AI state (Fig. 5C). The CBC data values have also been mentioned in the Supplementary Table S2. Initially, we investigated the effect of NH + GDP on LPS-induced serum IL-6 levels. As expected, NH + GDP markedly reduced serum IL-6 levels more than 30% (Fig. 5D) with suppressed JAK2 and STAT3 phosphorylation in liver (Fig. 5E). Consistent results were observed with decreased liver hepcidin-25 protein expression (Fig. 5F), thereby, down regulating IL-6/STAT3 pathway. Moreover, reduced liver *Hamp* expression indicates the increase in serum ferritin level for effective iron-mediated erythropoiesis thus, improving hypoferremia (Fig. 5G). Apart from liver, the spleen plays a significant role in chronic inflammation and immune response. Dysregulation of splenic iron is another hallmark of AI with reduced circulating iron level, thus we investigated the effects of NH + GDP on spleen during chronic AI. The tissue iron deposit and iron content of liver and spleen was reversed by NH + GDP reducing iron restrictive effect of inflammation with effective iron-mediated efflux (Fig. 5H,I). These data indicate that NH + GDP successfully ameliorates inflammatory hepcidin and improves AI symptoms in chronic model thus, maintaining the normal iron homeostasis

**Figure 6:**
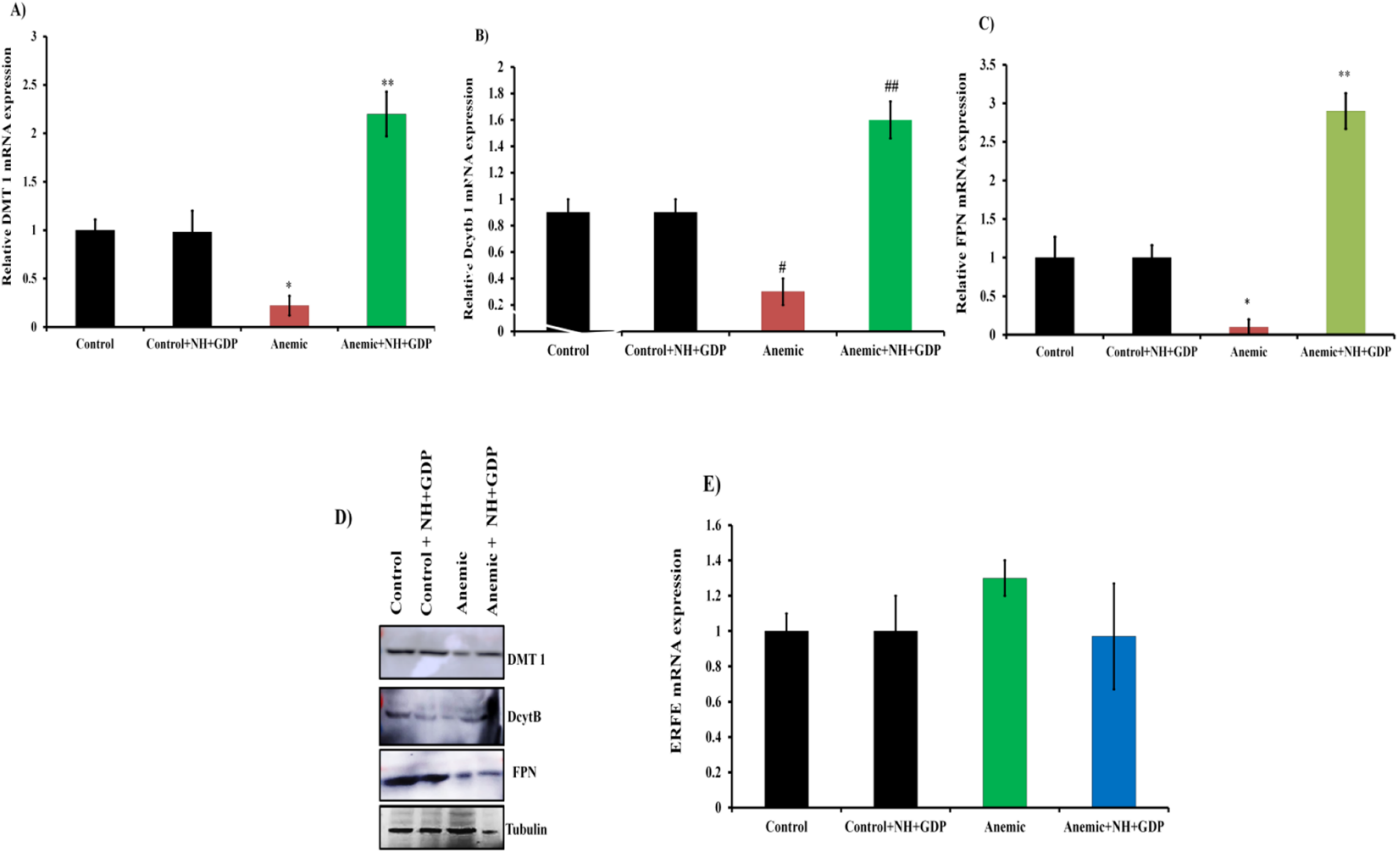
Effect of NH+GDP on intestinal gene (DMT1, DcytB and FPN) transporter in chronic mice model: (**A–D**) NH+GDP significantly increases the gene and protein expression of DMT 1, DcytB and FPN with effective iron egress as comparatively to anemic induced mice. *p* values were calculated using one-way ANOVA followed by Tukey’s post test. *^, #^*p <* 0.01; ***^, ##^*p <* 0.001, *p <* 0.05. (**E**) Gene expression analysis showed no significant increase in ERFE mRNA expression in comparison to anemic group.

To further elucidate the effect of NH + GDP on intestinal iron homeostasis, the mRNA and protein expression of DMT1, Dcytb and FPN were studied in the intestine of chronic mice model (LPS + Zymosan). In consistent with the earlier studies^26,27^ the DMT1 and FPN expression were found to be decreased in anemic mice due to hepcidin induced degradation of DMT1 as well as FPN channel. On NH + GDP treatment, increase DMT1 and FPN expression were observed with effective iron egress due to the suppressed hepcidin effect in comparison to anemic group. In parallel, as reported by previous study, the Dcytb repression was observed in anemic group due to the presence of inflammatory cytokines^28,29^. Whereas, NH + GDP treatment reverse this effect with increase Dcytb expression (Fig. 6A–D). These results indicate that NH + GDP was found to have significant role in recovering the intestinal iron transporters. In addition, the erythroferrone (ERFE) mRNA expression was studied in the spleen tissue of all the four different mice groups collected at 28 days. We found increased ERFE mRNA expression in the spleen but the increase was not significant which is in contradiction to the previous study where significant ERFE expression in spleen of BA (*Brucella abortus*)-induced AImice model was reported at 28 days^30^. It could be due to the fact that *Brucella abortus* elicits severe inflammatory response as compared to the Zymosan which was used in our study to induce AI mice model. Hence, it could be inferred that ERFE has no significant effect on the down regulation of hepcidin in chronic mice model. In addition, NH + GDP treatment showed no marked difference in the ERFE mRNA levels as compared to the anemic group (Fig. 6E).

## Discussion

Anemia of inflammation (AI) is the second most clinically prevalent anemia that greatly threatens public health with increase mortality and poor prognosis due to prolonged infection or inflammation^2,31^. Hepcidin, a hepatic origin peptide hormone, plays a crucial role in iron homeostasis^6^. Two major pathways regulating the activity of hepcidin are BMP/SMAD pathway particularly associated with body iron status and IL-6/STAT3 pathway regulating the inflammation associated hepcidin levels^5,8,32^. Many conventional remedies were developed for AI that include synthetic form of erythropoietin (EPO)^33^, blood transfusion and iron supplementation. Moreover, blood transfusion is associated with increased risk of disease transmission, immunomodulation and acute haemolytic reaction^34^. Additionally, oral iron supplementations are efficacious but poorly tolerated due to non-absorbed iron mediated gastrointestinal tract diseases^35^. These observations highlight the immediate requirement for alternative therapies and illustrate the significant implications of our findings in discovering a natural screened compound (GDP) with less toxicity and increase iron bioavailability in clinical management of AI.

Earlier studies have demonstrated the anti-inflammatory agents like hydrogen sulphide^16^, and AG490^19^ which attenuated hepcidin expression *in vitro* and *in vivo* experiments. However, their basic limitation was their stability, complex delivery mechanism, non-specific delivery and unclear metabolic profile. Since inflammation mediated hepcidin vastly contributes for AI, it should be taken into account that process of inflammation is initiated by resident macrophages which apart from releasing IL-6 also releases IL-1β, TNF-α and NO during inflammatory response^25^. TLR4, a type I trans membrane receptor, is activated by LPS and plays a significant role in innate immune system. LPS activate the TLR4-mediated signalling pathway that leads to the activation of NF-ҝB^23^. As reported earlier, 3, 4-dihydroxytoluene^36^, Resokaempferol^37^, chloroquonine^38,39 and Thymoquinone40^ inhibits NF-ҝB activation under inflammatory conditions, which is the major inducer of pro-inflammatory cytokines. Earlier we demonstrated that GDP acts as a novel hepcidin antagonist with dual role acting directly on hepcidin and indirectly attenuating IL-6/STAT3 pathways^21^. However, to explore the mechanistic aspects behind *Hamp* mRNA down regulation *via* IL-6/JAK/STAT3 pathway, we developed a liposomal drug delivery system (NH+) encapsulating GDP (NH + GDP) for investigating cellular iron efflux. In the present study, we found that NH + GDP inhibited the LPS-induced activation of NF-ҝB in U937 derived macrophages with suppressed expression of pro-inflammatory mediators. Herein, to identify the mechanistic action of NH + GDP involved in the inhibition of NF-ҝB activation signal, we investigated the effect of NH + GDP on LPS-induced U937 derived macrophages. NH + GDP was found to inhibit the LPS-induced phosphorylation and degradation of IҝB-α with reduced nuclear translocation of p65 NF-ҝB into the nucleus (Fig. 2B). In agreement with the decreased IL-6 levels in LPS-CM (Fig. 2C–D) subsequently there was reduced binding of IL-6 to IL-6 receptor attenuating the activation of JAK2/STAT3 pathway responsible for *Hamp* mRNA transcription in HepG2 cells (Fig. 3A,B). Though uncovering all the molecular networks related to signalling pathway is critical, we have only focused on LPS-induced NF-ҝB activation along with IL-6 mediated JAK2/STAT3 pathway which is one major contributor in the regulation of hepcidin levels. Though, we are not validating NH + GDP as an anti-inflammatory compound as it needs more experimental evidence. Indeed, for the first time, we established a link between NH + GDP inactivating NF-ҝB and IL-6/STAT3 pathway indicating an alternative approach for hepcidin modulation. Earlier, there was no such evidence in previous reports demonstrating that NH + GDP suppress NF-ҝB signalling attenuating IL-6/STAT3 pathway.

In current years, a remarkable attention on the ability to produce nanocarriers of uniform size, shape and composition have revolutionized the era of science and technology^30,41^. Liposomes for drug delivery system offer higher biocompatibility, versatility and lower toxicity as well as increases bioavailability and pharmacokinetics^22^. We investigated the cytotoxicity of the liposomal formulations (NH+ and NH++) at indicated concentration and found that comparative to NH+ formulation, NH++ is more toxic to the cell lines, as most of the cells surface is negatively charged thus, it has higher permeability for cationic charged particles, therefore double positive charge particles (NH++) penetrate the cell membrane more abruptly as compared to the particles with less positive or negative and neutral charge thus, resulting in cellular damage. In relevance to previous study^21^ *in vivo* results indicate that encapsulated GDP (NH + GDP) was more efficient and biocompatible than non-encapsulated (GDP) as relative serum iron concentration was increased by 52% due to its uniformity, stability and sustained drug release without any cellular toxicity. Next, we examined the effect of NH + GDP and GDP on IL-6 expression in U937 derived macrophage cells and found that relatively to GDP, encapsulated GDP (NH + GDP) was more effective in suppressing IL-6 level in dose dependent concentration. This assay marked compelling evidence that NH + GDP was more effective in reducing inflammation-mediated IL-6 secretion as compared to non-encapsulated GDP.

**Figure 7:**
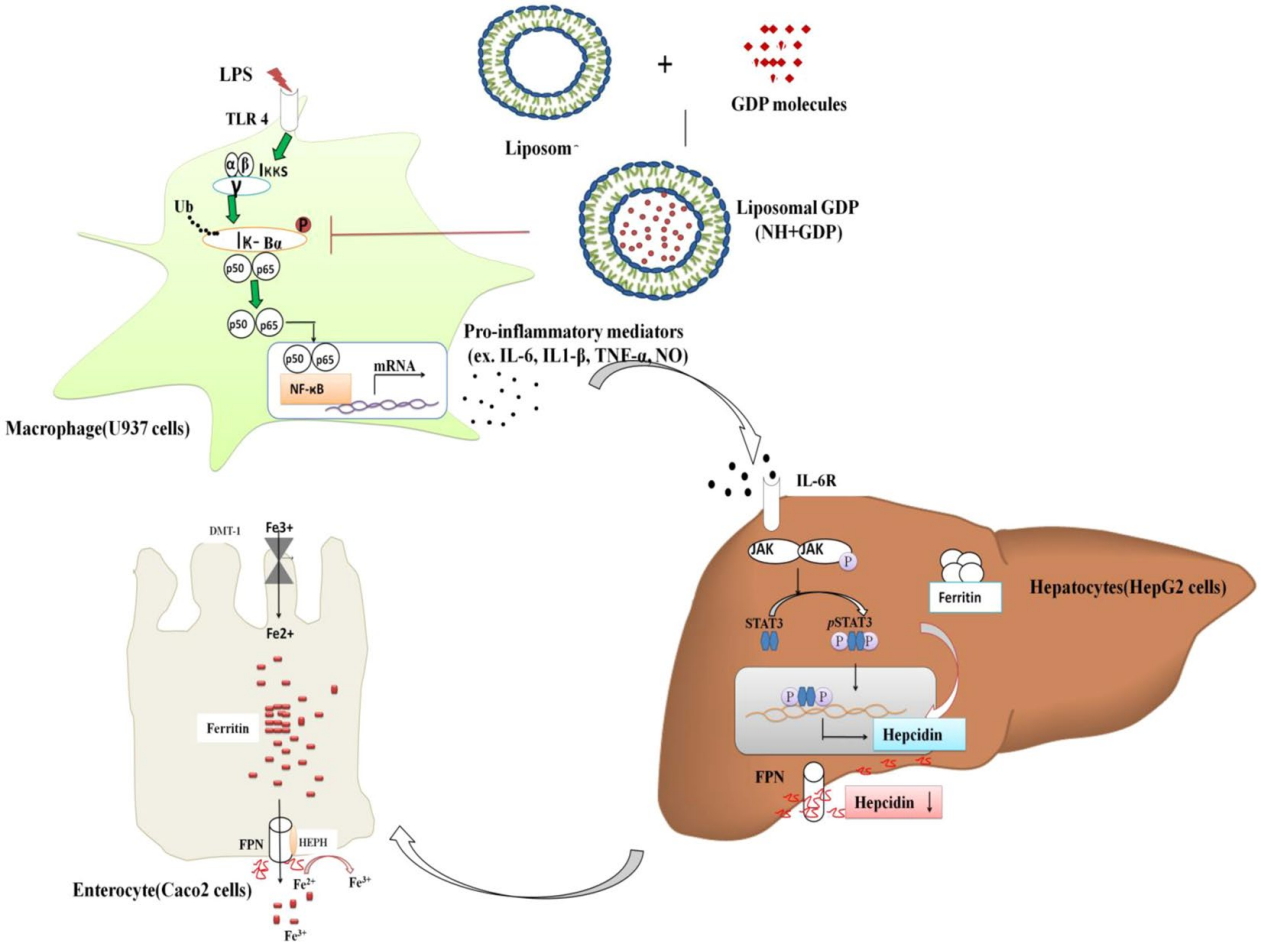
Graphical representation of the site of action of the liposomal (NH+GDP) on NF-ҝB pathway and IL-6/STAT3-hepcidin axis.

In the present study, we demonstrated the mechanism of NH + GDP attenuating NF-ҝB activation suppressing IL-6 secretion, which in turn decreases inflammation-mediated IL-6/JAK/STAT3 pathway (Fig. 7). Through this current study, we have shown its effect on the mechanistic NF-ҝB targeting IL-6/JAK/STAT3 pathway. Additionally, we encapsulated the GDP compound in the lipid vesicle to increase its stability, bioavailability and efficacy by using drug delivery system^42,43^. NF-ҝB is one of the most critical transcription factor that regulate the inflammation mediated gene expression in macrophage cells. We investigated whether IL-6, secreted from macrophages, is responsible for inflammation-mediated hepcidin induction through activation of JAK/STAT3 pathway. The CM model is a well-established model used in relevant studies as opposed to exposing hepatocytes to LPS induced inflammation^16,44^. STAT3 is a member of STAT family with its roles in cellular transformation, proliferation and metastasis of cancer^45^. IL-6 activated inflammatory pathway (JAK/STAT3) was focussed to examine the hepatic hepcidin expression which is linked to iron homeostasis. Furthermore, we aimed to develop the HepG2/Caco2 co-culture model by introducing HepG2 cells (expressing hepcidin) to the Caco-2 cells allowing epithelial interaction. For iron related co-transporter studies Caco-2 cell line is a well-established *in vitro* model. However hepatic peptide hormone hepcidin plays a significant role in response to inflammation by regulating cellular iron release^7^. Hepcidin produced in liver (HepG2) interacts with enterocytes (Caco2) and thereby decreases iron absorption by affecting the transport of iron and down regulates FPN expression^6^. We designed to develop the HepG2 model by introducing to Caco-2 cells. The HepG2 and Caco-2 epithelia were separated by a liquid compartment, which allowed for epithelial interaction^46^. We investigated how the LPS-CM induced hepatic epithelium (HepG2 Cells) interacts with the Caco-2 cells through the liquid compartment(Fig. 2A). The co-culture model is to predict the molecular mechanism of NH + GDP effect on inflammation induced IL-6/JAK2/STAT3-hepcidin pathway. In the HepG2 and Caco2 co-culture experiment using LPS-CM, we found decreased IL-6 secretion in CM which subsequently reduced its binding to the IL-6 receptor further attenuating the phosphorylation of JAK/STAT3 pathway resulting in decreased *Hamp* transcription in HepG2 cells (Fig. 3A–C). However, reduced *Hamp* transcription is reported with more effective iron egress (DMT-1 expression) in Caco2 cells^47^. Analogous results were obtained *in vivo* experiments. Hence, the studied effect of NH + GDP has unrolled a novel and potential therapy against AI (Fig. 7).

Concomitantly, NH + GDP in acute model provide a compelling evidence of suppressed IL-6 levels along with down regulated JAK/STAT3 pathway. Next, our target was to evaluate the effect of NH + GDP on LPS + Zymosan induced chronic model in overcoming AI and to maintain normal iron homeostasis. AI is responsible for decreased iron binding ferritin levels (hypoferremia), with consequent iron restricted erythropoiesis; therefore a number of infectious and inflammatory mice and rats model have been established till date^48–50^. To induce chronic AI, many well-known models have been established to study the effect of inflammation on iron regulation and erythropoiesis in mice and humans such as turpentine^20,51^, LPS + Zymosan^52^, and complete Freund’s adjuvant^53^. In the present study, we used LPS + Zymosan induced chronic AI model to test the hypothesis that NH + GDP suppresses inflammation-mediated NF-ҝB and IL-6/JAK2/STAT3 pathway activation. The results of the current study agree with previous data showing that hepcidin inversely correlates with the levels of intestinal iron transporters whereas on NH + GDP treatment the iron transporters of intestine was found to be maintained, thus, impacting the intestinal iron homeostasis as well. Our results demonstrate that NH + GDP reverses acute inflammatory hepcidin and hypoferremia in chronic AI model with increase in haemoglobin level for effective iron mediated erythropoiesis thus, maintaining normal iron homeostasis.

## Conclusion

We demonstrated for the first time, that NH + GDP inhibits the release of pro-inflammatory mediators by suppressing NF-ҝB and JAK2/STAT3 activation in macrophages and hepatocytes respectively thus, decreasing hepcidin levels. Further, these findings reveal that NH + GDP could be a novel therapeutic agent to overcome the limitations associated with current therapies in inflammation-induced anemic conditions and suggest being a potential drug to relief AI.

## Materials and methodology

### Preparation and Characterization of liposomes formulations

Liposomal formulations were prepared by the thin film hydration method^42,43,54^ with some modifications.Briefly,(1,2-dioleoyl-3-trimethylammonium–propane(chloridesalt;DOTAP),1,2-dioleoyl-sn-glycero-3-phosphoethanolamaine–N-Lactosyl(LacPE),1,2-Dioleoyl-sn-glycero-3-phosphoethanolamine (DOPE) purchased from Avanti polar lipids InC were dissolved in chloroform solutions (Sigma Aldrich USA, 100 mg/ml) and mixed at the desired molar ratios (Table 1). The organic solvent was then evaporated by rotary evaporation to obtain a lipid film. Later, the film was hydrated with 2 ml of PBS at 100 μM GDP. The resulting lipid suspensions were then vigorously shaken, and the liposomes obtained were homogenized by means of an extruder (Avanti polar lipex) through 2 stacked polycarbonate membranes (0.1 μm pore size, Avanti Polar Lipids) to finally obtain uni-lamellar liposomes. The particle size distribution and zeta potential (ζ) of the final liposomal formulations were measured by DLS using a Zetasizer Nano ZS (Malvern Instruments, UK).

### Encapsulation efficiency

Encapsulation efficiency (EE) of liposomes was determined using the dialysis technique for separating the non-captured drug from liposomes. Liposome GDP (100 µL) was diluted in 900 µL ethanol, and then it was exposed to sonication until complete liposomes disruption. The supernatant was collected, and the absorbance was measured by UV spectrophotometry^55,56^. The measurement was carried out in triplicate and EE of the liposomes was determined as in Equation 1.

$${\rm{EE}}( \% )=\{({{\rm{C}}}_{{\rm{i}}}\mbox{--}{{\rm{C}}}_{{\rm{f}}})/{{\rm{C}}}_{{\rm{i}}}\}\times 100$$

C_i_: Initial concentration of drug used in formulating the liposomes

C_f_: Concentration of drug in the supernatant.

### Morphological studies

Transmission Electron Microscopy (TEM) and Scanning Electron Microscopy (SEM) were used to characterize the morphology of the liposome. For TEM (JEOL 2100), the samples were prepared by applying a drop of the mixture to a carbon-coated copper grid, and left for one minute to allow some of the particles to stick to the carbon substrate. After removing the excess of dispersion with a piece of filter paper a drop of 1% phosphotungstic acid solution was applied and left to air-dry. For SEM (Hitachi S-4300SE/N) special treatment was required to dry up the wet samples. Air-drying of samples causes damage due to evaporation with reduced surface tension therefore, critical point drying was performed to avoid such damage before observing at different magnification.

### *In vitro* release study of encapsulated drug (NH + GDP)

The *in vitro* release study of GDP from NH+ was carried out at 37^0^C under magnetic stirring using dialysis bag (mol.wt. cut-off-12 kDa). For *in vitro* drug release experiment briefly, 2 ml of liposomal GDP solution was kept in a dialysis membrane and dialyzed against 20 ml PBS buffer solution (pH-6.8). At a regular interval of time, 1 ml of the medium was withdrawn and the drug content was determined using UV spectrophotometer at 253 nm. The same volume of fresh fluid was used to replenish the release medium. The release test was continued for 12 h. The cumulative fraction of release GDP versus time was expressed by following equation:

$${\rm{Cumulative}}\,{\rm{release}}\,{\rm{GDP}}( \% )={[{\rm{GDP}}]}_{{\rm{t}}}/{[{\rm{GDP}}]}_{{\rm{total}}}\times 100$$

Where [GDP]_t_ is the amount of GDP released at time t, [GDP]_total_ is the total GDP present in the GDP-loaded liposomes.

### Cytotoxicity assays

Cell viability assays (MTT) were simultaneously performed using two cell lines (HepG2 and Caco2). Cells were seeded in 6 well plate at 0.25 × 10^5^cells/well^57,58^. The cells were treated with different liposome formulation and detailed experimental setup is provided in supplementary data. Before treatment to the cells, medium was removed and fresh supplemented medium containing liposome formulations (NH+, NH++ 100, 1000 μg) and encapsulated (NH + GDP and NH++ GDP) at indicated LPS concentration (10, 20, 30, 40 and 50 μg/ml) was added and incubated for 24 h.

Non-treated cells were used as 100% viability control (dotted line). All the measurements were done in triplicate in three independent experiments. Dose-response curves were fitted using a sigmoid dose-response curve model provided in the Graph Pad Prism 5.0 (Graph Pad software, USA). Differences among data were analysed using One-way ANOVA followed by Tukey’s post-test *p* < 0.005.

### Cell culture

The Caco2 and HepG2 cell lines (obtained from NCCS, Pune, India) were cultured at 37 °C in humidified air (95%) and 5% CO_2_ in Dulbecco Modified Eagle Medium (DMEM), supplemented with 1% (v/v) antibiotic solution and 10% FBS (Thermo Fisher Scientific, Invitrogen, USA). The medium was changed every alternate day, and cells were passaged at approximately 75% confluence. Before treatment, cells were seeded in 6 well plates at an optimum density of 0.3 × 10^6^ cells/well and confluent at 1.2 × 10^6^ cells/well. U937, a human monocytic cell line was maintained in RPMI 1640 medium with 10% FBS and 1% penicillin and streptomycin and were incubated with PMA (100 nM)^16^ for overnight to induce differentiation to macrophages. Before treatment, U937 derived macrophages cells were cultured in fresh medium, followed by LPS stimulation (1 μg/ml) for 6 h and treatment with NH + GDP for 1 h. The cell-free supernatant of the culture medium was collected as conditioned medium (CM), which was defined as “pre-treatment”. The HepG2 cells inserts were transferred to the Caco2 wells and both cell lines were incubated together in LPS-CM. Further the LPS-CM was exposed to the HepG2 and Caco2 co-culture model, termed as “post treatment”. Moreover, for the mono-culture study with IL-6 as a stimulant, HepG2 and Caco2 cells were exposed IL-6 (10 ng/ml) for 6 h followed by harvesting of cells for further studies.

### Internalization of encapsulated MANT-NH + GDP fluorescence signals in HepG2 and Caco2 cells

The Caco2 and HepG2 cells were treated with encapsulated NH + MANT (2’-(or-3’)-*O*-(*N*-Methylanthraniloyl)-GDP (10 μM Thermo Fisher Scientific, Invitrogen, USA) a fluorescent analogue of GDP and incubated for 24 h. Images were acquired using Leica Inverted microscope.

### Nitric oxide measurement

Cells were seeded at 1 × 10^5^cells/well in 24-well plate. U937 derived macrophages cells were pre-treated with LPS stimulation in a dose dependent manner for 24 h followed by NH + GDP treatment for 30 min. After 24 h the supernatant (100 μl) was collected and further it was incubated with 1% sulphanilamide solution (50 μl) for 10 min in dark. Later 0.1% NED solution (50 μl) was added and absorbance was measured at 550 nm.The concentration of NO was calculated using NaNO_2_ standard curve. Data are representative of three independent experiments.

### Quantitative gene expression studies

Total RNA was isolated from cell lines (Caco2, HepG2) and from tissue sample (liver, intestine) using RNA extraction kit (Invitrogen, USA). The qualitative ratio metric analysis of RNA was done using Infinite 200 PRO Nano Quant (Tecan, Switzerland). RNA (2 µg) was reverse transcribed to single strand cDNA using cDNA synthesis kit (High capacity reverse transcription kit, Applied Bio system). Quantitative gene expression of *Hamp*, TNF-α, IL-1β, IL-6 was quantified by qRT-PCR (Applied Bio systems 7500 Fast Real-Time PCR machine), using gene specific primers (Supporting Information Table S1). Data was analysed using ΔΔC_t_ method (Biosciences, Qiagen, USA). Glyceraldehyde-3-phosphate dehydrogenase (GAPDH) was used as an internal control for normalization of quantitative gene expression data.

### Animal experimentation

Male BALB/c mice weighing between 25–30 g were procured from the Central Animal House of Panjab University, Chandigarh. The animals were housed in polypropylene cages bedded with sterilized husk. They were given free access to clean drinking water (tap water *ad libitum*) and standard animal pellet diet (Ashirwad Industries, Kharar Punjab, India), throughout the experiment. Animals were acclimatized to laboratory conditions before the experiment. The experimental protocols were approved by the Institutional Ethics Committee and conducted according to Indian National Science Academy Guidelines for the use and care of experimental animals.

### Acute LPS model

To evaluate the effects of NH + GDP on hepatic IL-6/STAT3 pathway and hepcidin expression during acute inflammation, BALB/c mice were intraperitoneally (IP) pre-injected with NH + GDP (30 mg/kg body weight)^21^. Several studies have shown that hepcidin induction is maximal at 6 h time point in response to LPS^59–61^. Thirty minutes after the last injection, mice were challenged with 0.1 mg/kg^62^ bodyweight LPS (IP) to induce inflammation and sacrificed 6 h later after anaesthesia with pentobarbital sodium. Normal saline was used as vehicle control. Animals were randomly divided into four groups (n = 8 group each). Control, Control + NH + GDP, LPS model, LPS + NH + GDP. Blood was drawn and tissues were isolated and stored at −80 °C. All the experimental procedures were conducted at the end of the experiment.

### Chronic LPS + Zymosan A model ameliorating AI

For chronic model, BALB/c mice were IP injected with a single dose of LPS (0.1 mg/kg body weight) followed a week later by an injection of Zymosan A as 0.8 mg/g body weight (a preparation from yeast wall). Chronic inflammation was induced by IP injection of 40 μg LPS diluted in 200 μL saline solution, followed by an I.P injection of Zymosan A (16 mg diluted in 500 μL saline)^52^ for two week for induction of anemia. After 16 days, mice were then treated with NH + GDP (IP i.e. 30 mg/kg body weight)^21^ for two weeks and later sacrificed. Animals were randomly divided into four groups (n = 8 group each). Control, Control + NH + GDP, Anemic, Anemic + NH + GDP. Control animals were administered with normal saline for next two weeks. Tissues were isolated and stored at –80 °C for further studies.

### Hepcidin and ferritin measurement

Total cellular protein was extracted using Radio Immunoprecipitation assay (RIPA) buffer (Sigma-Aldrich, USA) and a protease inhibitor cocktail (Sigma-Aldrich, USA). The hepcidin (YH biosearch laboratory (YB B20150924152) and ferritin ICL antibodies (E-90F) level were determined using ELISA as per user manufacture instruction. The results were normalized for the total protein concentration in each sample. Protein concentration was determined using the bicinchoninic acid assay (BCA assay, Thermo Fisher Scientific, Invitrogen, USA).

### Immunofluorescence staining

U937 cells were fixed with 4% formaldehyde as described^20^. FITC tagged goat anti-rabbit IgG (Sigma Aldrich USA) were used as the secondary antibody and was diluted in PBS with 0.1% Tween 20 and incubated for 1 h in the dark, followed by staining with Hoechst (Invitrogen, USA) for another 10 min. The slides were observed by Leica Inverted microscope.

### Western blotting

Cellular protein and tissue protein extract were prepared and western blotting was performed^21^. The extracted proteins were transferred to PVDF membranes (Invitrogen, USA) and after washing with TBST, membrane were blocked with 2% bovine serum albumin and further incubated with primary antibodies.

Antibodies used are: pIKBα, IKBα, NFκB, pNFκB (cell signalling technology Danvers MA, USA)

Mouse anti-ferritin antibody (1:1000; Sigma-Aldrich, USA)

Rabbit anti-SLC-40A1 antibody (1:1000; Sigma-Aldrich, USA)

Rabbit anti-phospho STAT3 antibody (1:1000, Sigma-Aldrich, USA)

Rabbit anti TNF-α antibody (1:5000; Sigma-Aldrich, USA)

Rabbit anti-IL-1β antibody (1:1000; Sigma-Aldrich USA)

Rabbit anti-DMT1 antibody (1:1000; Sigma-Aldrich USA)

Rabbit anti-hepcidin antibody (1:1000; Sigma-Aldrich USA)^63,64^

Rabbit anti-JAK2 antibody (1:1000; Sigma-Aldrich, USA)

Rabbit anti-p-JAK2 antibody (1:1000; Sigma-Aldrich, USA)

Rabbit anti-STAT3 antibody (1:1000; Sigma-Aldrich, USA)

Rabbit CYBRD1 antibody (1:1000; Sigma-Aldrich, USA)

Further the blot was incubated with secondary peroxidase-labelled antibodies, anti-rabbit IgG antibody (1:5000; Sigma-Aldrich USA) and anti-mouse IgG antibody (1:5000; Sigma-Aldrich USA) for 1 h. Anti-*α*-tubulin antibody was used as a loading control. PVDF membrane for immune-reactive band was detected by chemiluminesence reagent (Sigma, USA) and Immunostaining kit (Sigma-Aldrich, USA). Immunoblot signals were captured using the Amersham Image 600. Immunoblot were quantified using Image J software, and band densities were normalized with the corresponding tubulin band densities.

### Serum iron and intracellular iron measurement

Serum was separated immediately by centrifugation of the blood samples for 15 min at 4 °C. Serumand spleen tissue samples were used for acid digestion in the microwave accelerated reactor system (MARS6, CEM Corporation, USA). Intracellular iron concentration and iron contents of organs (liver, spleen) were estimated in the digested samples using inductively coupled plasma mass spectrometry (ICP-MS; 7700 × AgilentTechnologies, Santa Clara, CA).

### Tissue histological analysis

Tissue histology sections were obtained for each sample, fixed in 4% formaldehyde and embedded in paraffin. Thereafter, sections were stained with Perl’s Prussian blue to visualize iron deposits. Images were acquired using Leica Laser-Tech (GmbH, Heidelberg, Germany).

### Hematologic parameter measurement

Blood was obtained by retro-orbital phlebotomy of mice and collected in heparinized tubes. Erythrocyte parameters were determined using double chamber Haematology Auto-analyser (BC-3000 plus).

### Statistical analysis

Statistical differences between qRT-PCR data were analysed using one-way analysis of variance (ANOVA). Subsequently, Tukey’s multiple comparison procedure was employed to check the level of significance using Prism Graph Pad software (Graph Pad Software Inc, San Diego, CA, USA). The experiments were carried out in triplicate to calculate standard deviation (SD), standard error of mean (SEM) and level of significance (based on *p* value). In all the tests, *p* ≤ 0.05, *p* ≤ 0.01and *p* ≤ 0.001 was taken as the criterion for statistical significance.

## Electronic supplementary material

Supplementary information
