# Supplementary material for: Novel Liposome Eencapsulated Guanosine Di Phosphate based Therapeutic Target against Anemia of Inflammation
Source: Sci Rep. 2018 Dec 6;8:17684. doi: 10.1038/s41598-018-35992-2 (PMC6283875; doi:10.1038/s41598-018-35992-2)
Supplement: Supplementary file 1 — Supplementary information [file 41598_2018_35992_MOESM1_ESM.pdf]

Novel Liposome Eencapsulated Guanosine Di Phosphate based Therapeutic Target against Anemia of Inflammation

Stanzin Angmo[1], Shilpa Rana[1], Kamalendra Yadav[1], Rajat Sandhir[2], and Nitin Kumar Singhal[1*]

[1]Food Science and Technology Department, National Agri-Food Biotechnology Institute (NABI)

Sector-81(Knowledge City), PO Manauli, S.A.S. Nagar, Mohali-140306, Punjab, India.

[2]Department of Biochemistry, PanjabUniversity-160014, Chandigarh, India.

Running Title: Encapsulated GDP (NH+GDP) down regulates NF-κB/IL-6/STAT3 mediated hepcidin expression.

Corresponding Authors:

Dr.Nitin Singhal PhD, Food Science and Technology Department, National Agri-Food Biotechnology Institute (NABI)

(An autonomous Institute of Department of Biotechnology, Govt. of India)Sector-81 (Knowledge City), PO Manauli, S.A.S. Nagar, Mohali -140306, Punjab, India.

Tel: 0172-5221243

E-mail: nitin@nabi.res.in

Abbreviations: AG490-Tyrphostin B42(JAK2 inhibitor), AMPK- Adenosine 5'-monophosphate-activated protein kinase , H_2S-Hydrogen sulfide, SIRT1- Sirtuin 1, SPRC- S-propargyl-cysteine, TLR-Toll like receptors, IL-1 –Interleukin 1, BMP-Bone Morphogenetic proteins, JAK-Janus kinase, IL-6-Interleukin-6, TNF-α-Tumor Necrosis Factor alpha, STAT-Signal Transducer and activator of transcription, MTT-3-(4,5-dimethylthiazol-2-yl)-2,5-diphenyl tetrazolium bromide, CM-Conditional medium.

Supplementary Figures

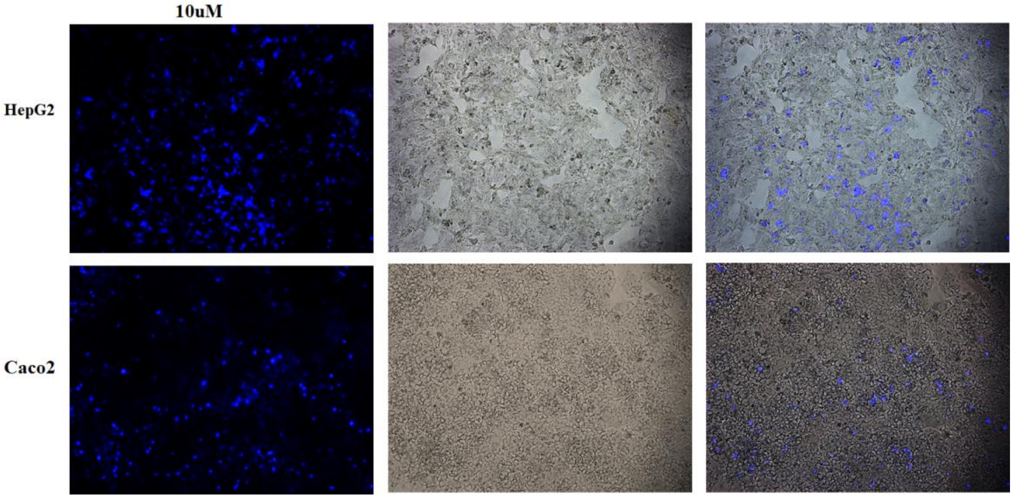

Figure S1: Internalization of MANT-NH+GDP in HepG2 and Caco2 cell: Internalization of encapsulated NH+MANT (2'-(or-3')-*O*-(*N*-Methylanthraniloyl)-GDP (10µM) with merge bright field image .

In vitro release study of encapsulated drug (NH+GDP)

The release rate of the NH+GDP was measured at 37°C in PBS at pH-6.8 for over a period of twelve hours and the measurement was taken at 253 nm. The value of concentration corresponding to the absorbance was calculated from the GDP standard curve.The release profile of GDP from NH+ showed that 78.98% of the drug was released in 6 h, followed by a steady release rate. The release behaviour of GDP from the NH+ liposomal formulation exhibited a biphasic pattern characterized by a steady and continuous release for a period of 12 h.

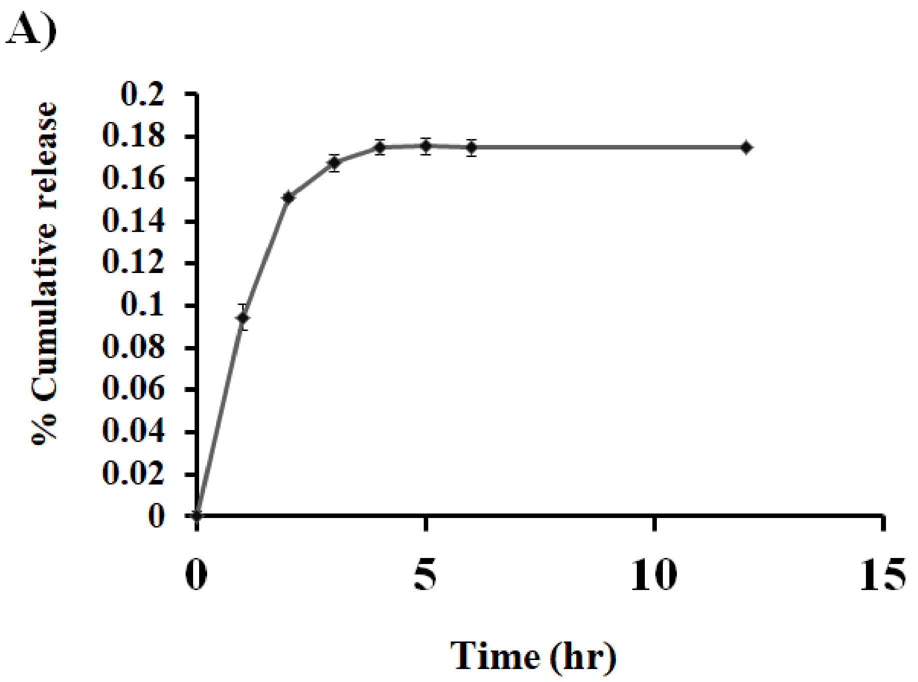

Figure S2: A) Release profile of encapsulated NH+GDP at different time interval at pH6.8.

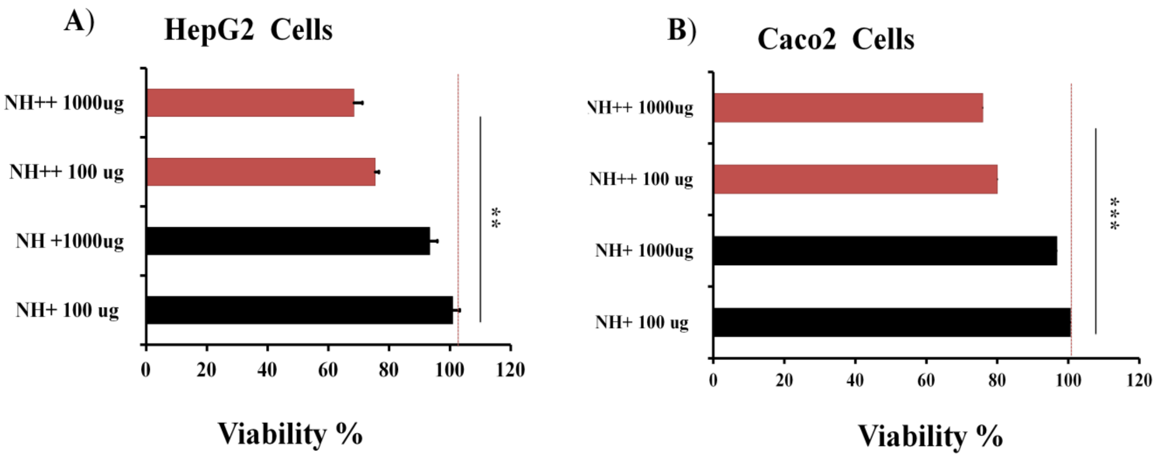

Figure S3: Evaluation of Cytotoxicity of cationic liposomes (NH+ and NH++) without encapsulation on HepG2 and Caco2 cells. A-B) Viability of liposome formulation (NH+ and NH++) was determined using MTT assay at different concentration indicating NH++ more toxic than NH+ on both HepG2 and Caco2 cells. Data represent means ± SD of three independent experiments. Differences were analyzed using One-way ANOVA followed by Tukey's post test. (**) $p< 0.05$; (***) $p < 0.001$.

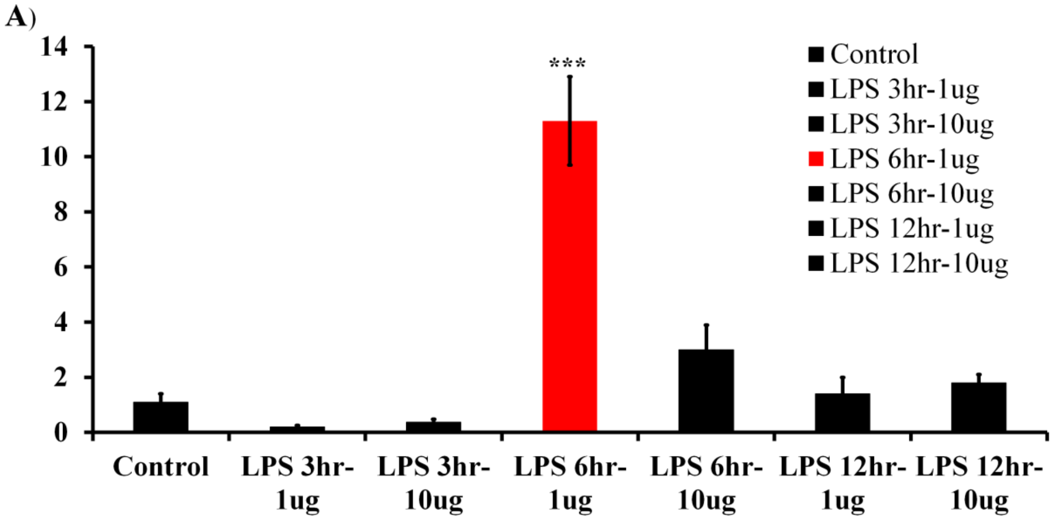

Figure S4: Dose dependent concentration of LPS at different time interval on HepG2 cells: A) The HepG2 cells were treated with LPS at indicated concentration at different time intervals. Time course evaluation suggested that increase in *Hamp*mRNA level (12 fold) was observed at LPS (6h-1µg) in HepG2 cells. Data represent means ± SD of three independent experiments. Differences were analyzed using One-way ANOVA followed by Tukey's post test. (***), *p*< 0.001.

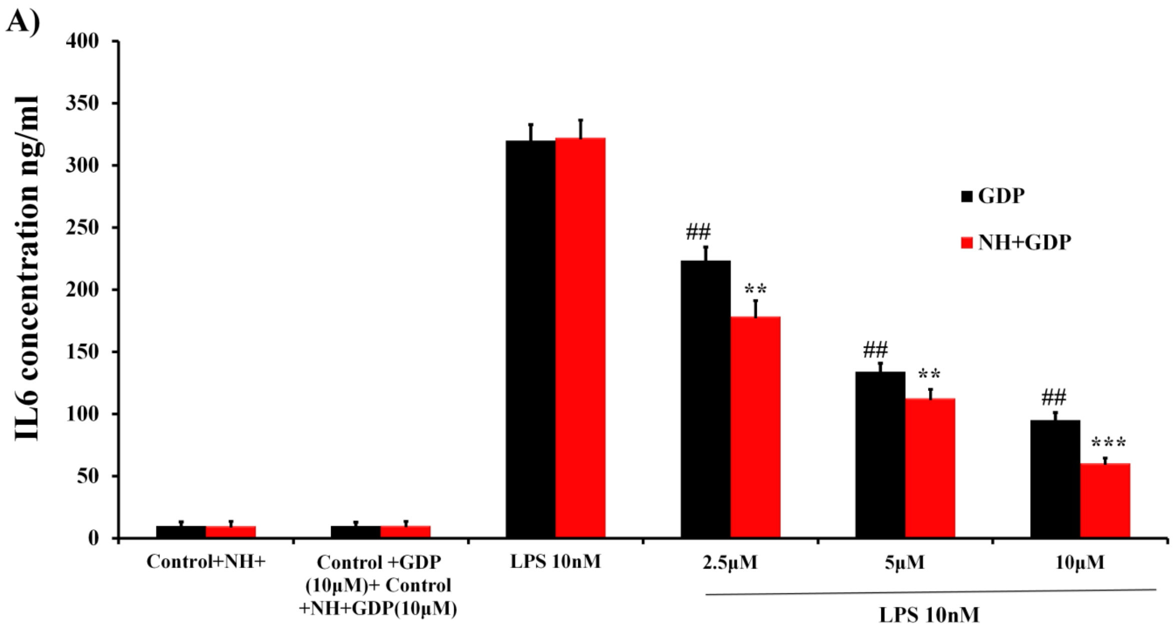

Figure S5: Effect of encapsulated (NH+GDP) and non-encapsulated (GDP) on IL-6 level:

LPS-induced inflammation elevates IL-6 level in dose dependent concentration. In comparison to non-encapsulated (GDP), NH+GDP was more effective in suppressing IL-6 level in U937 macrophages cell. *p* values were calculated using one-way ANOVA. **: $p \leq 0.05$,##:$p \leq 0.01$, (***) $p < 0.001$.

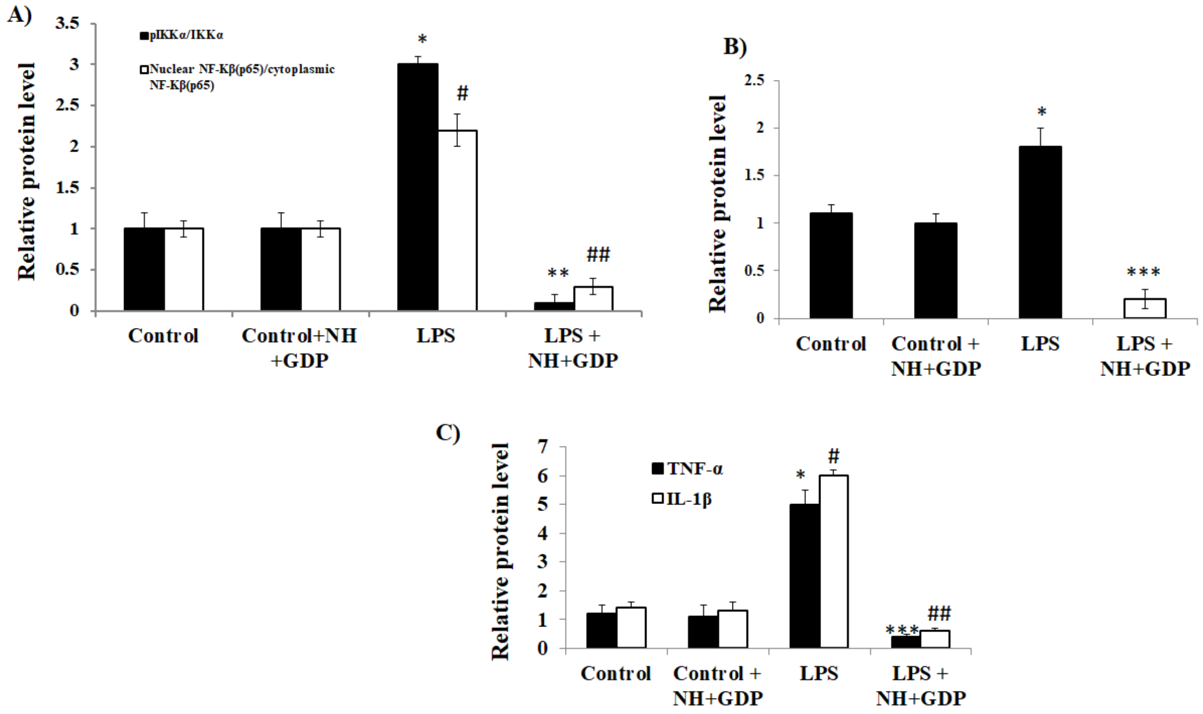

Figure S6: NH+GDP suppresses NF-κB activation decreasing pro-inflammatory cytokine inductionin U937 macrophages cells: A-C) Densitometry analysis of immunoblot were scanned to quantify the level of pIKKα, nuclear NF-κB(p65)/cytoplasmic NF-κB(p65), IL-6,TNF-α and IL-1β relative to tubulin densities. Differences were analyzed using One-way ANOVA followed by Tukey's post test. (*, #) $p < 0.01$; (***) ;$p < 0.001$, (**,##);$p < 0.05$.

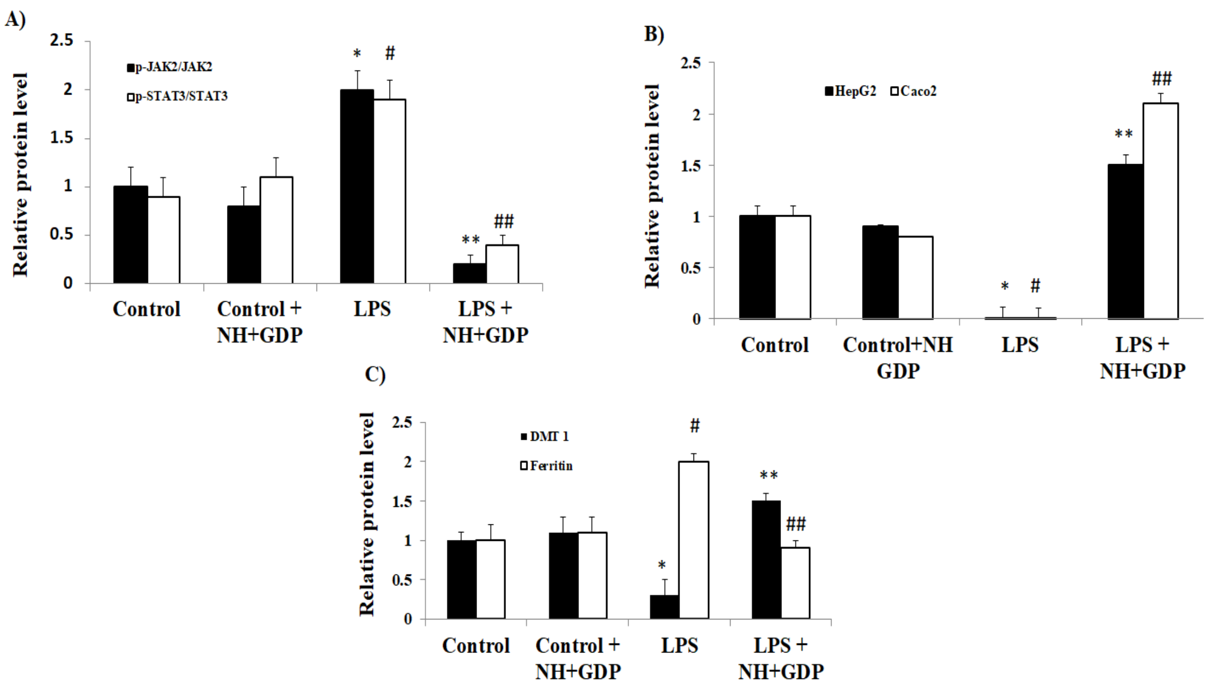

Figure S7: NH+GDP attenuates IL-6 secretion with suppressed JAK2/STAT3 phosphorylation: (A-C) Immunoblots were scanned and densiometric analysis was used to quantify the level of hepatic JAK2/STAT3 phosphorylation ,FPN,DMT1 and ferritin level in HepG2 and Caco2 coculture cellrelative to their tubulin densities.Data represent means ± SD of three independent experiments. Differences were analyzed using One-way ANOVA followed by Tukey's post test. (*, #) $p < 0.01$; (**##) $p < 0.05$.

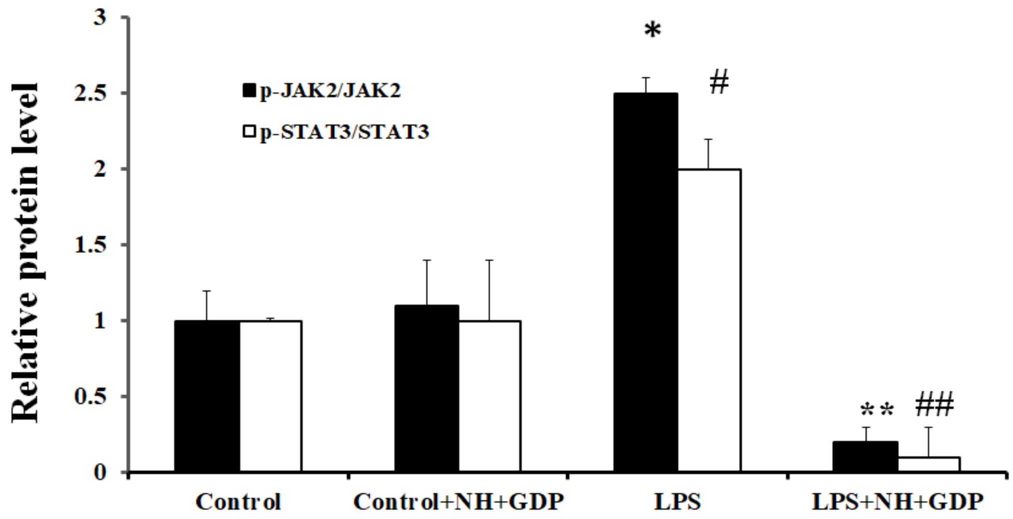

Figure S8: NH+GDP suppresses IL-6/JAK/STAT3 activation in acute BALB/c mice model: (A) Densitometry analysis of hepatic JAK2/STAT3 phosphorylation relative to tubulin densities.Data represent means ± SD of three independent experiments. Differences were analyzed using One-way ANOVA followed by Tukey's post test; (*, #) $p < 0.01$; (**, ##) $p < 0.05$.

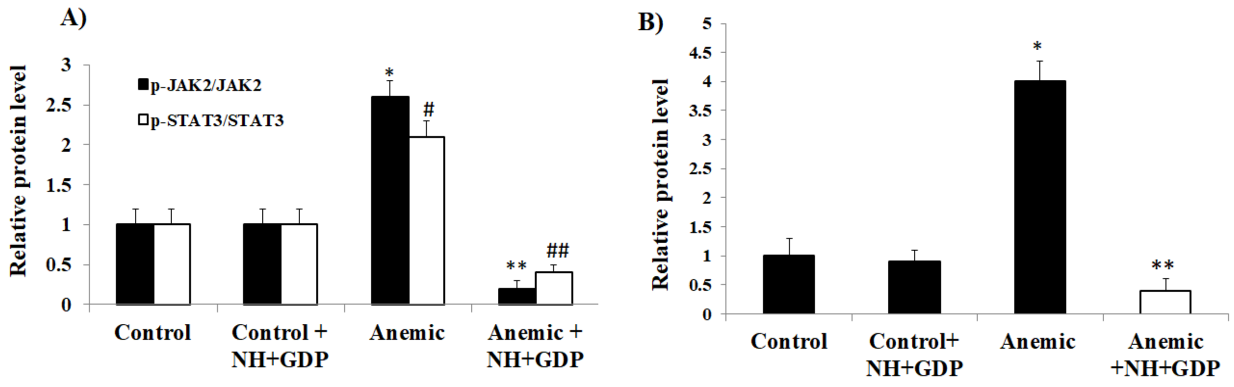

Figure S9: NH+GDP suppresses IL-6/STAT3 pathway in chronic AI model: A-B) Immunoblots were scanned and densiometric analysis was used to quantify the level of, hepatic JAK2/STAT3 phosphorylation and *Hamp*levelin hepatocytes relative to tubulin densities. Data represent means ± SD of three independent experiments. Differences were analyzed using One-way ANOVA followed by Tukey's post test. (*, #) $p < 0.01$; (**, ##) $p < 0.05$.

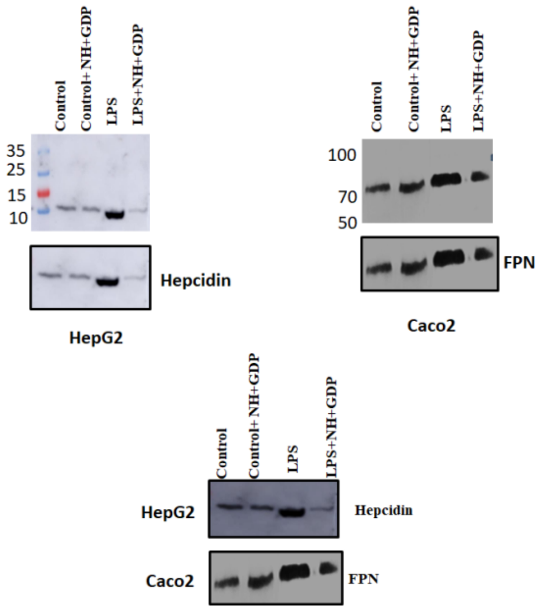

Figure S10: Effect of IL6 in monoculture (HepG2 and Caco2 cells): A) IL-6 stimulated HepG2 monoculture cells showed decrease in hepcidin protein expression in NH+GDP treated

group. **B)** In IL-6 stimulated Caco2 monoculture cells no marked difference in FPN expression was observed in LPS treated group as compared to control.

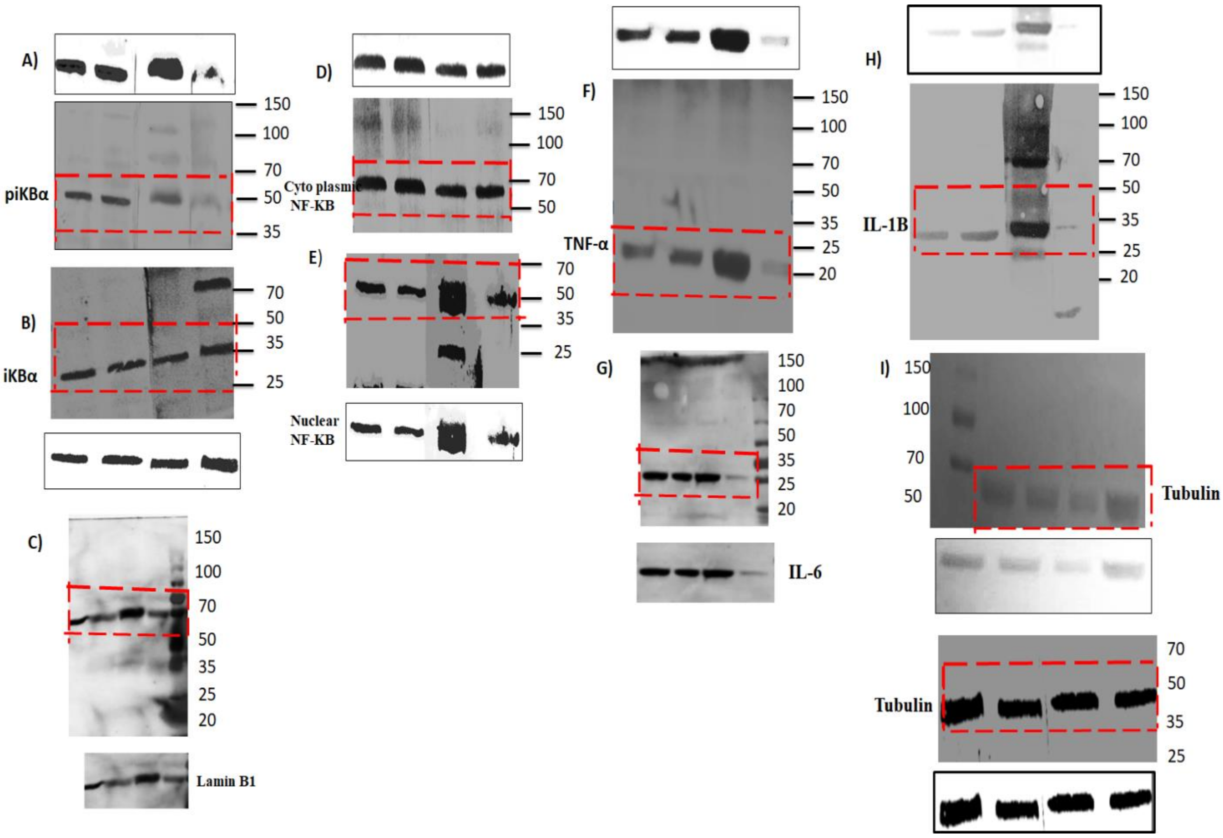

Supplementary Figure S11: **A-B)** The U937 cell lysates were processed for phosphorylation of IκB-α(pIκB-α) later the blots was processed and then stripped and re-stained with IκB-α antibodies (presented in crop version). **C-D)** NH+GDP prevents the nuclear translocation of the p65 subunit of NF-κB from cytosol into the nucleus, later the blots was stripped and re-stained with nuclear NF-κB antibodies(presented in crop version). **E-F)** Treatment with NH+GDP significantly decreases protein expression in TNF-α , later the blots was processed in parallel and then stripped and re-stained with IL-6 antibodies(presented in crop version). **G-H)** The U937 cell lysates were processed for TNF-α and tubulin and later the blots were then stripped and re-stained with anti-tubulin antibodies,(presented in crop version). Relevant molecular markers used are (Page Ruler Prestained Protein Ladder, Thermo Scientific).

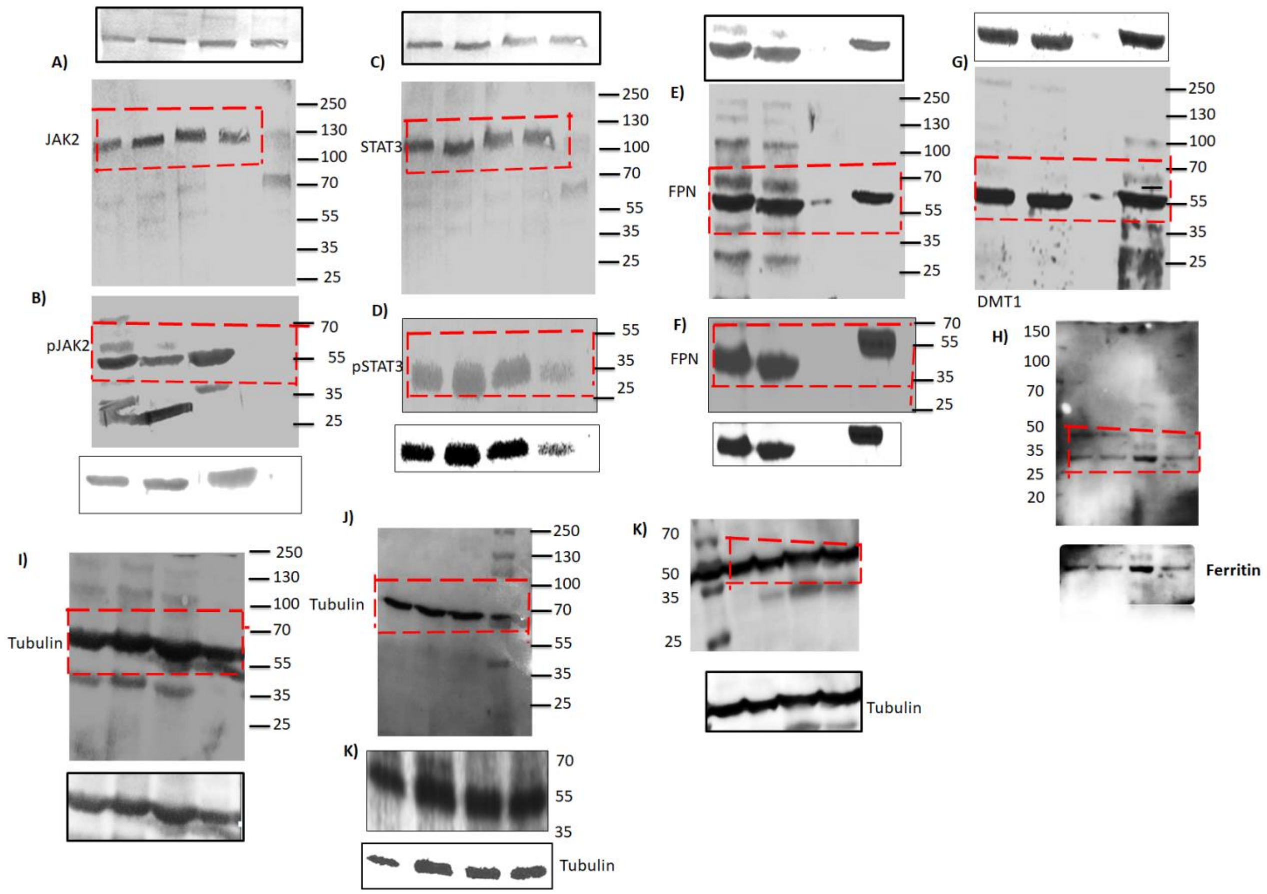

Supplementary Figure S12: **A-B)** The LPS-CM-induced HepG2 cell were processed for JAK2 activation and later the blots was processed and then stripped and re-stained with pJAK2 antibodies (presented in crop version). **C-D)** LPS-CM-induced HepG2 lysates were also processed for STAT3 activation, and the blots was processed and then stripped and re-stained with pSTAT3 antibodies (presented in crop version). **E-F)** Western blotting of the lysates of HepG2 and Caco2 were processed for FPN expression; later the blots were stripped and re-stained with FPN antibodies (presented in crop version). **G-H**) The LPS-CM-induced HepG2/Caco2 co-culture model was processed for evaluating DMT1 expression in Caco2 cells, and later the blots was processed and then stripped and re-stained with ferritin antibodies (presented in crop version). **I-J)** The LPS-CM-induced HepG2 cells were processed for tubulin(I,J) and later the blots were then stripped and re-stained with anti-tubilin antibodies,

(presented in crop version, **K)** Relevant molecular markers used are (Page Ruler Prestained Protein Ladder, Thermo Scientific).

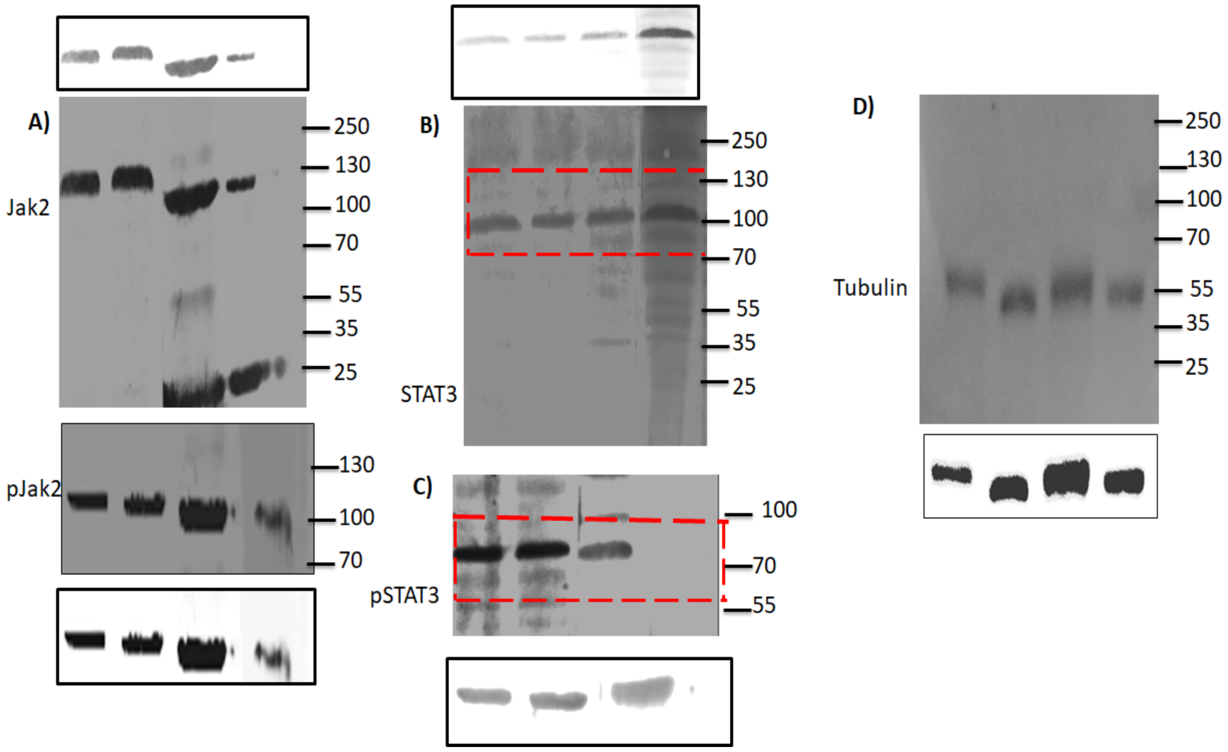

Supplementary Figure S13: **A-B)** The treated NH+GDP hepatocytes(liver) cells were processed for JAK2 activation and later the blots was processed and then stripped and re-stained with pJAK2 antibodies (presented in crop version). **C-D)**NH+GDP hepatocytes (liver) were also processed for STAT3 activation, and the blots was processed and then stripped and re-stained with pSTAT3 antibodies (presented in crop version). **E)** Blot presents tubulin as loading control. Relevant molecular markers used are (Page Ruler Prestained Protein Ladder, Thermo Scientific).

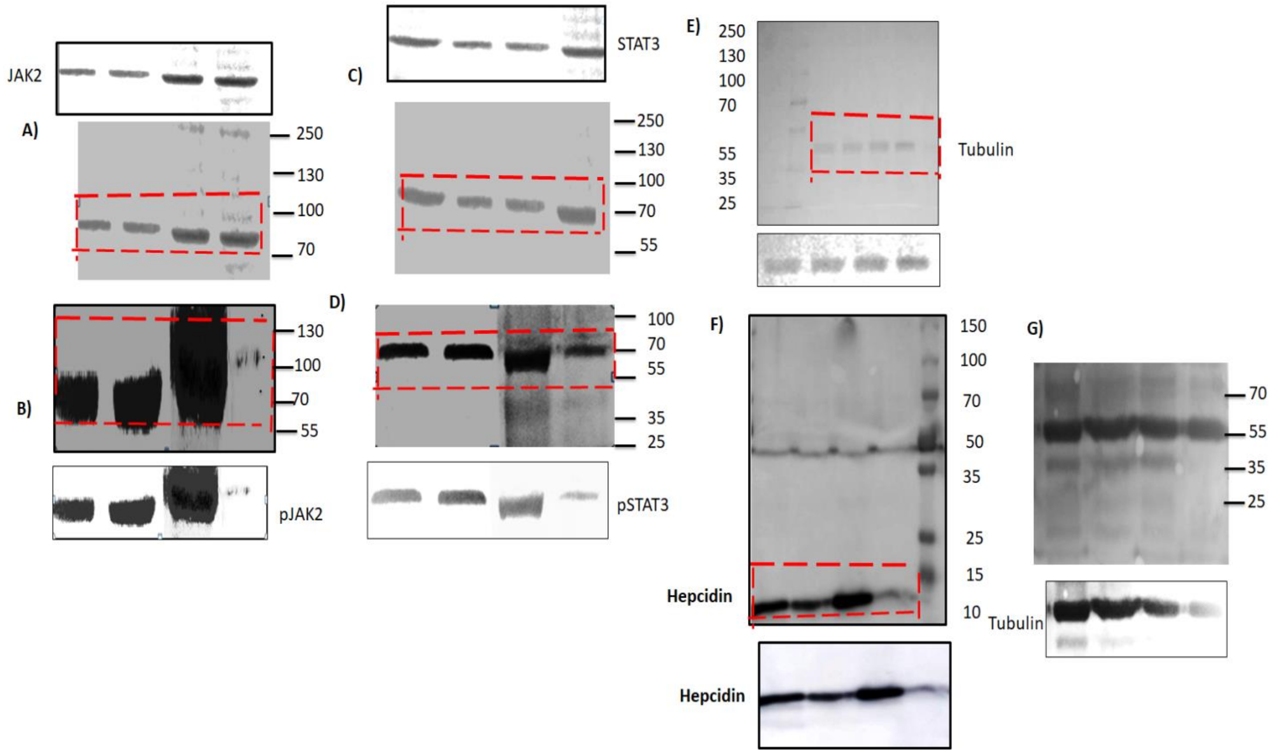

Supplementary Figure S14: **A-B)** The treated NH+GDP hepatocytes(liver) cells in chronic mice model were processed for JAK2 activation and then stripped and re-stained with pJAK2 antibodies (presented in crop version). **C-D)** NH+GDP hepatocytes (liver) were also processed for STAT3 activation, and the blots was processed and then stripped and re-stained with pSTAT3 antibodies (presented in crop version). **E)** Blot presents tubulin as loading control. **F-G**) Blot represents hepcidin-25 and tubulin as loading control. Relevant molecular markers used is (Page Ruler Prestained Protein Ladder, Thermo Scientific).

Supplementary Tables

Table S1: List of primer for semi quantitative RT-PCR.

**Human and mouse target gene**	**Primer(5'- 3')**
**H GAPDH-F**	**5′-GAAGGTGAAGGTCGGAGTC-3′**
**H GAPDH-R**	**5′-GAAGATGGTGATGGGATTTC-3′s**
**H *Hamp*-F**	**5′-CTCTGTTTTCCCACAACAGAC-3′**
**H *Hamp*-R**	**5 ′-TAGGGGAAGTGGGTGTCTC-3′**
**H IL-1β-F**	**5 ′-AGCCATGGCAGAAGTACCT-3′**
**H IL-1β-R**	**5 ′-CAGCTCTCTTTAGGAAGACAC-3′**
**H TNF-α-F**	**5 ′-TCTCGAACCCCGAGTGACAA-3′**
**H TNF-α-R**	**5 ′-TGAAGAGGACCTGGGAGTAG-3′**
**H IL6-F**	**5 ′-TCGAGCCCACCGGGAACGAA-3′**
**H IL6-R**	**5 ′-GTGGCTGTCTGTGTGGGGCG-3′**
**m GAPDH-F**	**5'- GTGGAGATTGTTGCCATCAACGA-3'**
**m GAPDH-R**	**5'-CCCATTCTCGGCCTTGACTGT-3'**
**m *Hamp*-F**	**5'-GGCACTCAGCACTCGGACCCA-3'**
**m *Hamp*-R**	**5'-TTGGTATCGCAATGTCTGCCCTGC-3'**

Table S2: Complete blood count (CBC)indices of mice injected (I.P) with normal saline or LPS+Zymosan and treated with NH+GDP (*i.p.* i.e. 30 mg/kg body) for 2 week.

Hematological parameters	Control	Control + NH+GDP	Anemic	Anemic + NH+GDP
**Hb (g/dl)**	**16.7 ± 0.34**	**15.8 ± 1.02**	**11.2 ± 0.11** [a]	**15.4 ±1.44** [a]
**MCV (fL)**	**49.35 ± 0.45**	**47.2 ± 0.55**	**42.60 ±1.433** [b]	**48.6 ± 0.36** [b]
**MCH (pg)**	**17.00 ± 1.55**	**15.00 ± 0.22**	**14.9 ±0.54** [c]	**16.9 ± 1.55** [c]
**RBCs (10^{12} /L)**	**09.18 ± 1.33**	**09.00 ±0.33**	**7.625 ±1.0** [d]	**09.03 ± 0.28** [d]
**Platelets (10^{9} /L)**	**1203 ± 0.551**	**1098 ±1.466**	**1954 ±2.56** [e]	**2728 ± 0.71** [e]

Values are expressed as mean ± SD; *n* = 5

[a, b, c ,d,e] Anemic + NH+GDP is highly significantly from Anemic. (*p* < 0.05)
